# EDCC-RPL: A novel energy-efficient and load-balanced objective function for RPL Routing in IoT networks enhancing network lifetime and reliability

**DOI:** 10.1371/journal.pone.0346827

**Published:** 2026-04-21

**Authors:** Muhammad Asif Habib, Abdullah M. Albarrak, Mudassar Ahmad, Alaa E. S. Ahmed, Naeem Raza, Hafiz Muhammad Sajid Imran, Muhammad Abdul Qayum, Habib Ur Rahman

**Affiliations:** 1 College of Computer and Information Sciences, Imam Mohammad Ibn Saud Islamic University (IMSIU), Riyadh, Saudi Arabia; 2 Department of Computer Science, National Textile University, Faisalabad, Pakistan; 3 Department of Computer Science, National University of Modern Languages, Faisalabad, Pakistan; 4 Department of Software Engineering, The University of Lahore, Lahore, Pakistan; PLOS: Public Library of Science, UNITED KINGDOM OF GREAT BRITAIN AND NORTHERN IRELAND

## Abstract

Energy efficiency and balanced load distribution remain persistent challenges in the Routing Protocol for Low-Power and Lossy Networks (RPL), significantly affecting the lifetime and reliability of Internet of Things (IoT) deployments. This study introduces EDCC-RPL, a novel multi-metric objective function that integrates Expected Transmission Count (ETX), end-to-end delay, and child count through an adaptive additive model. Unlike conventional single or dual metric schemes such as OF0, MRHOF, and EA-EPL, EDCC-RPL simultaneously enhances energy efficiency, network stability, and scalability. Extensive simulations in Contiki Cooja with 20–50 nodes demonstrate up to 32% lower energy consumption, 18% higher Packet Delivery Ratio (PDR), and 50–60% reduction in parent switching (churn) in dense topologies. These improvements validate EDCC-RPL’s novelty in achieving joint optimization of reliability, delay, and load balancing. The proposed approach provides a practical and scalable solution for sustainable IoT networks in smart cities, industrial monitoring, and environmental sensing applications. The implementation code of the proposed EDCC-RPL algorithm is publicly available at https://github.com/drmasifhabib/edcc-rpl-github for reproducibility and reuse.

## Introduction

**Fig 1:**
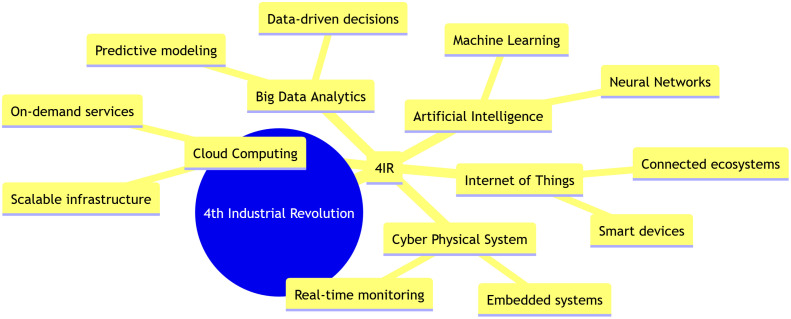
Industry 4.0 Technologies.

In Industry 4.0 environments, such as smart manufacturing and industrial automation, Internet of Things (IoT) networks form the backbone of real-time monitoring, predictive maintenance, and autonomous control systems [1]. These environments typically rely on dense deployments of resource-constrained sensor and actuator nodes that are required to operate continuously with minimal human intervention. Energy-efficient routing is therefore a critical requirement, as frequent battery depletion or node failures can disrupt industrial processes, reduce system reliability, and increase operational costs. Moreover, industrial IoT applications impose strict requirements on latency, reliability, and stable network operation to support time-sensitive control loops and data-driven decision making. Inefficient routing mechanisms often lead to uneven energy consumption, congestion near sink nodes, and shortened network lifetime, directly undermining the scalability and sustainability goals of Industry 4.0 systems. These challenges motivate the need for energy-aware and load-balanced routing solutions, such as the proposed EDCC-RPL objective function. Industry 4.0 involves integrating humans into the manufacturing process, emphasizing continuous improvement, value-adding activities, energy savings, and waste reduction. The concept of Industry 4.0 remains visionary and is driven by technologies such as CPS (Cyber-Physical System), Cloud Computing, Big Data and Analytics, and the Internet of Things (IoT). Industry 4.0 represents the fourth industrial revolution, characterized by the integration of cyber-physical systems, the Internet of Things, and cloud computing into manufacturing environments. This transformation enables autonomous decision-making and real-time communication between machines, products, and humans, leading to highly efficient “smart factories” [2]. Fig 1 illustrates the technologies associated with Industry 4.0. These technologies serve as the main pillars of Industry 4.0. Among them, IoT covers a wide range of applications in daily life, including smart homes, smart agriculture, and the construction industry.

**Fig 2:**
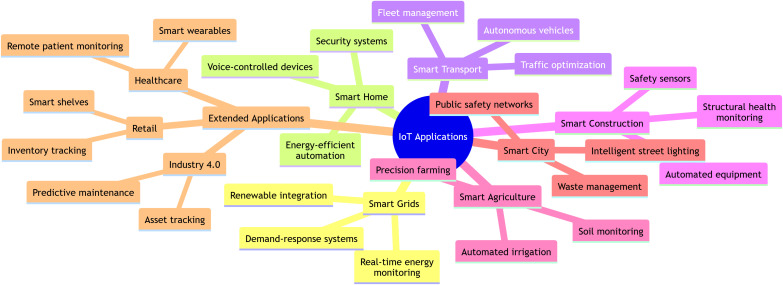
Applications of IoT.

The applications of IoT are illustrated in Fig 2. The acronym IoT derives from the words “Internet” and “Things.” The Internet enables connectivity among various technological devices, living organisms (including animals, humans, and birds), and non-living objects (such as clothing and food). The word “of” links “Internet” with “Things,” highlighting ubiquity. The Internet of Things (IoT) connects physical objects via embedded sensors and software, allowing data exchange across networks for automated control and remote monitoring. Its applications span a range of fields, including industrial automation, smart cities, healthcare, and precision agriculture, leading to significanth improvements in operational efficiency and resource management [3].

**Fig 3:**
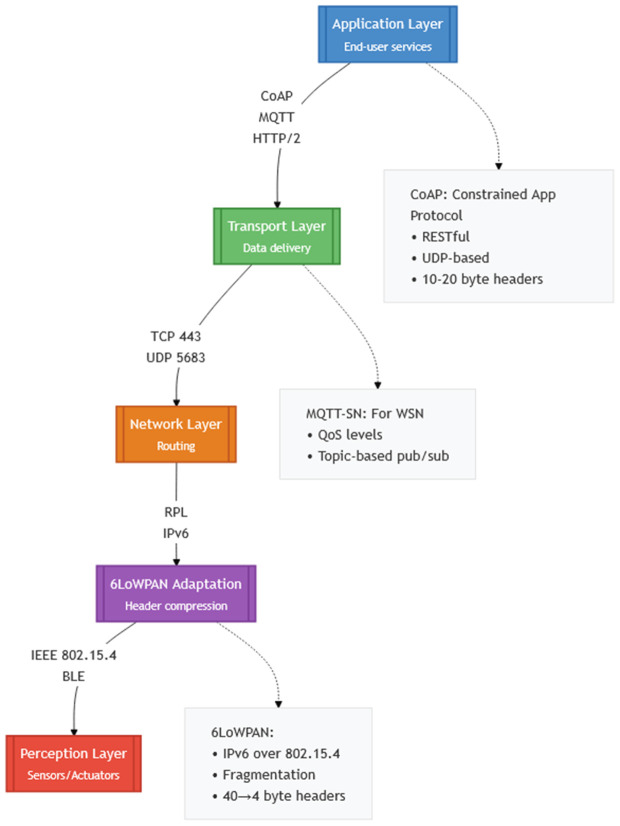
IoT Protocol Stack.

The IoT protocol stack defines its functionalities. Each IoT layer uses different communication protocols to support various operations. The detailed protocol stack of IoT is shown in Fig 3. IoT mainly depends on two key application protocols: Message Queuing Telemetry Transport (MQTT) and Constrained Application Protocol (CoAP). Protocols like UDP and Transmission Control Protocol are part of the transport layer. Routing Protocol for Low Power and Lossy Networks (RPL) and IPv6 over Low Power Wireless Personal Area Networks (6LoWPAN) function at the network layer. The functionality of the IoT perception layer is supported by IEEE 802.15. This layer also includes sensors connected to gather data from different sources. IoT ecosystems rely on foundational technologies like RFID, wireless sensor networks, and communication protocols (e.g., MQTT, CoAP) to establish scalable, low-power connectivity among diverse devices. These technologies enable transformative use cases in environmental sensing, smart transportation, and personalized healthcare, reshaping how humans interact with the physical world [4]. Energy-efficient and stable routing in IoT networks has a direct and measurable impact on cost savings and operational reliability in several real-world application domains. In smart grid infrastructures, reliable and energy-aware routing ensures continuous monitoring of power distribution assets, reducing maintenance costs and preventing outages caused by node failures. In healthcare IoT systems, where wearable and implantable devices are often battery-powered, stable routing minimizes communication disruptions and extends device lifetime, thereby improving patient safety and reducing replacement and maintenance expenses. Similarly, in smart factories, predictive maintenance applications rely on uninterrupted data collection from large numbers of sensors deployed across production lines. Inefficient routing in such environments leads to premature energy depletion, network instability, and increased downtime, which directly translates into higher operational costs and reduced productivity. Consequently, energy-efficient and load-balanced routing mechanisms are essential for maintaining reliability and cost-effectiveness in large-scale industrial and mission-critical IoT deployments [5].

**Fig 4:**
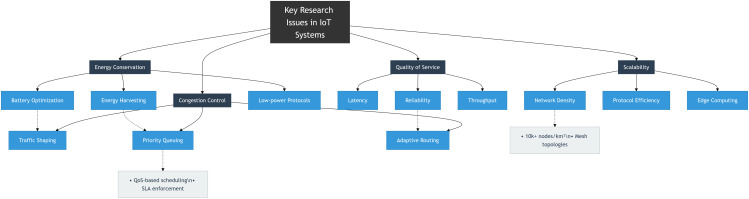
Key Research Issues in IoT Systems.

Implementing IoT in practice presents various challenges to the research community, including energy conservation, quality of service, security, and network connectivity. These challenges are depicted in Fig 4. Our proposed EDCC-RPL algorithm directly tackles the energy conservation issue, aiming to enhance the performance and energy efficiency of IoT routing protocols. Scalability in IoT refers to the system’s ability to add new devices and services without impacting current performance. The wide range of device capabilities presents significant challenges, requiring a scalable architecture. While cloud computing offers centralized scalability, edge computing allows for local processing. However, it lacks historical data storage and advanced analytics, as seamless device integration and dynamic topology management demand additional infrastructure and resources. Quality of Service (QoS) in IoT involves maintaining performance metrics such as latency, bandwidth, and throughput across different applications. Managing these parameters is especially critical in sectors like healthcare. Several strategies, including optimization algorithms, machine learning, and blockchain, have been suggested. Still, their effectiveness is complicated by the ever-changing and diverse nature of IoT environments.

Congestion control is becoming increasingly vital as device density and data traffic grow. Traditional TCP methods are unsuitable for constrained IoT devices, so lightweight, adaptive protocols are necessary. Cross-layer strategies spanning MAC, network, and application layers combined with AI techniques like reinforcement learning, enable predictive control and improved network efficiency. Energy conservation remains a major challenge in IoT because devices often need to operate for extended periods with limited power. Standard protocols such as HTTP and TCP are inefficient for low-power systems. Therefore, lightweight communication stacks, energy-aware MAC protocols, and optimized routing are crucial. Achieving the right balance between performance and power efficiency is key for sustainable deployment. Recent research highlights the limitations of the RPL protocol, the standard for IoT routing. Its Route Path Management (RPM) faces issues such as slow convergence, uneven load distribution, sub-optimal path selection, and high control overhead. These problems result in poor energy usage and shorter network lifespans. At the application layer, CoAP’s energy-intensive update mechanism, along with the lack of adaptive scheduling at the MAC layer, further diminishes energy efficiency and performance. Comparison of parent selection strategies in RPL-based IoT routing is summarized in Table 1. Impact of energy-efficient and stable IoT routing on real-world applications is summarized in Table 2.

**Table 1:** Comparison of parent selection strategies in RPL-based IoT routing.

Approach Type	Metrics Used	Strengths	Limitations in Dense IoT
Single-metric	Hop count or ETX	Low complexity, fast computation	No load awareness, congestion near sink, rapid energy drain
Dual-metric	ETX + Energy / Delay	Improved reliability or latency	Partial optimization, weak load balancing
Proposed multi metric (EDCC RPL)	ETX + Delay + Child Count	Reliable links, low latency, balanced load	Requires weight tuning (addressed via adaptive design)

**Table 2:** Impact of energy-efficient and stable IoT routing on real-world applications.

Application Domain	Role of IoT Routing	Impact of Energy-Efficient & Stable Routing
Smart Grids	Continuous monitoring of meters, sub stations, and grid assets	Reduced maintenance cost, fewer outages, extended node lifetime
Healthcare IoT	Wearable and medical sensor data transmission	Improved reliability, longer device lifetime, enhanced patient safety
Smart Factories (Predictive Maintenance)	Real-time equipment health monitoring	Reduced downtime, lower operational cost, improved productivity

This paper introduces EDCC-RPL, a novel objective function for the Routing Protocol for Low-Power and Lossy Networks (RPL). The proposed approach combines Expected Transmission Count (ETX), end-to-end delay, and child count metrics using an additive composite method with adjustable weights.

Recent research continues to improve RPL-based IoT routing through cross-layer optimization and intelligent metric adaptation. For instance, an adaptive congestion-aware routing model enhancing energy and delay performance in dense IoT topologies, and an artificial-intelligence-driven objective function selection mechanism for dynamic environments were introduced. Similarly, a machine-learning-based parent selection strategy that minimizes route churn and improves reliability, and developed a composite metric formulation for energy-balanced and delay-constrained IoT communications were also introduced. Along with these many potential applications of IoT in recent times are also highlighted in [6–9]. These recent works provide significant insights into RPL enhancement; however, most either rely on static metric weighting or lack integrated load-awareness. Building on these gaps, the present study proposes EDCC-RPL, which adaptively combines ETX, delay, and child count metrics to ensure balanced load distribution, energy efficiency, and network stability in large-scale IoT deployments. Although the Routing Protocol for Low-Power and Lossy Networks (RPL) is widely adopted in IoT systems, its existing objective functions exhibit fundamental limitations in dense and large-scale deployments. Objective Function Zero (OF0) relies primarily on hop count–based rank computation and does not incorporate load-awareness, causing traffic concentration near preferred parents and rapid energy depletion of nodes close to the sink [10]. The Minimum Rank with Hysteresis Objective Function (MRHOF), while improving link reliability through ETX-based routing, similarly lacks mechanisms for load balancing and congestion mitigation, which leads to suboptimal performance under high node density [11].

Energy-aware extensions such as EA-EPL partially address energy considerations; however, they fail to jointly manage load distribution and latency, resulting in uneven energy consumption and accelerated node exhaustion in dense IoT networks. These limitations reduce overall network lifetime and hinder the scalability of IoT systems, highlighting the need for a load-balanced and energy-efficient routing strategy such as the proposed EDCC-RPL objective function. Parent selection in RPL is inherently a multi-criteria decision problem, particularly in dense and dynamic IoT environments. Single-metric objective functions, such as hop count or ETX alone, optimize only one aspect of routing and often lead to unintended consequences, including congestion, uneven energy depletion, or increased latency. Dual-metric approaches partially mitigate these issues; however, they still fail to capture the complex trade-offs among link reliability, timeliness, and load distribution. In this work, Expected Transmission Count (ETX) is employed to ensure reliable link selection, delay is incorporated to satisfy latency-sensitive application requirements, and child count is used as a load-awareness indicator to prevent parent congestion and traffic hotspots. The joint consideration of these three complementary metrics enables balanced parent selection, reduces energy drain on heavily loaded nodes, and enhances overall network stability. This design choice is particularly critical in the context of future IoT ecosystems, which are projected to scale to thousands or even millions of interconnected devices across smart cities, industrial automation, and large-scale sensing infrastructures. In such ultra-dense deployments, inefficient routing decisions can rapidly accelerate energy depletion, increase control overhead, and shorten network lifetime. Consequently, efficient, scalable, and load-balanced routing mechanisms are not merely desirable but essential for ensuring the sustainability and long-term operability of next-generation IoT networks [5].

**Fig 5:**
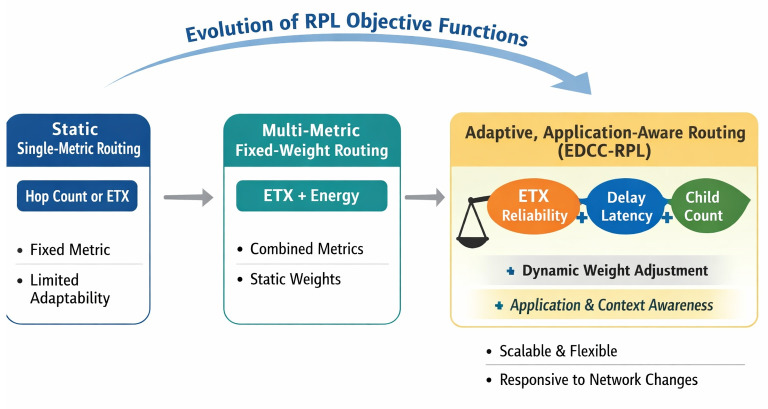
Conceptual evolution of RPL objective functions from static single-metric routing to adaptive, application-aware routing enabled by EDCC-RPL.

Sustainable smart city infrastructures increasingly rely on intelligent and adaptive networking solutions to reduce energy consumption, maintenance costs, and environmental impact. Recent studies emphasize the integration of learning-based and optimization-driven approaches to enhance the sustainability of IoT-enabled urban systems [12]. In this context, energy-efficient and load-aware routing mechanisms such as EDCC-RPL play a crucial role in supporting long-term, scalable, and sustainable smart city deployments. Beyond performance improvements, the proposed EDCC-RPL objective function represents a step toward adaptive and application-aware IoT routing. Unlike conventional RPL objective functions that rely on static or single-purpose metrics, EDCC-RPL jointly considers link reliability (ETX), latency (delay), and load distribution (child count) through a weighted composite design. This structure allows the routing behavior to be tuned according to application-specific requirements, such as prioritizing low latency for time-critical services or balanced load distribution for long-term energy sustainability. Moreover, by accommodating dynamic variations in network density, traffic patterns, and node conditions, EDCC-RPL enables routing decisions to evolve with changing environments. This adaptability makes EDCC-RPL a foundational building block for future intelligent IoT routing solutions that can seamlessly support heterogeneous applications and dynamic operational contexts. Fig 5 represents the evolution of RPL routing toward daptive and application-aware IoT routing. This diagram is illustrating the progression from Static single-metric routing (e.g., hop count, ETX), Multi-metric but fixed-weight routing (e.g., ETX + energy), and Adaptive, application-aware routing (EDCC-RPL) by integrating ETX, delay, and child count with tunable weights responding to network dynamics and application needs.

The main contributions of this paper are summarized as follows:

1. Novel multi-metric RPL objective function: We propose EDCC-RPL, a new objective function for RPL that jointly integrates Expected Transmission Count (ETX), end-to-end delay, and child count into a unified composite metric, enabling reliable, low-latency, and load-balanced parent selection in IoT networks.
2. Load-aware and energy-efficient routing design: By explicitly incorporating child count as a load-awareness indicator, the proposed approach mitigates parent congestion, balances traffic distribution, and reduces uneven energy depletion, thereby extending overall network lifetime.
3. Adaptive and application-aware routing capability: EDCC-RPL supports adjustable metric weighting, allowing routing behavior to be tuned according to application requirements and dynamic network conditions, positioning it as a step toward adaptive and context-aware IoT routing.
4. Comprehensive simulation-based evaluation: The proposed objective function is implemented and evaluated using the Contiki Cooja simulator across varying network densities, and its performance is compared against benchmark RPL objective functions (OF0, MRHOF, and EA-EPL) using key metrics such as energy consumption, packet delivery ratio, packet loss rate, churn, and hop count.
5. Demonstrated suitability for large-scale and future IoT deployments: Extensive results show that EDCC-RPL achieves improved energy efficiency, stability, and scalability, making it suitable for dense and large-scale IoT scenarios envisioned in Industry 4.0 and beyond.

### Design and Implementation of a New Objective Function

Development of EDCC-RPL, which uses an additive composite approach to combine multiple metrics Expected Transmission Count (ETX), end-to-end delay, and child count for better parent node selection. This addresses limitations in standard RPL objective functions like OF0 (Objective Function Zero) and MRHOF (Minimum Rank with Hysteresis Objective Function).

### Critical analysis of metric combinations

In-depth evaluation of how ETX, delay, and child count interact across various IoT applications, demonstrating improved load balancing, reduced congestion, and enhanced energy efficiency in constrained networks.

### Comprehensive simulation-based evaluation

Conducted extensive experiments using the Contiki Cooja simulator with random topologies and varying node densities (20 − 50 nodes). The study compares EDCC-RPL against OF0, MRHOF, and EA-EPL across key performance metrics including power consumption, Packet Delivery Ratio (PDR), packet loss rate, network stability (measured by average churn), and average hop count. Results show superior performance, with up to 32% reduction in energy use, 18% higher PDR, and 50 − 60% lower churn in dense scenarios.These contributions enhance RPL routing by promoting sustainable IoT deployments, with future prospects for real-testbed validation and dynamic weight optimization for application-specific tuning.

### Focus and motivation of the study

The primary focus of this research is to enhance the efficiency and stability of IoT routing by addressing the persistent issues of energy depletion, load imbalance, and congestion in the Routing Protocol for Low-Power and Lossy Networks (RPL). Existing objective functions, such as OF0 and MRHOF, often fail to ensure uniform load distribution and suffer from frequent parent switching, leading to early node failures and reduced network lifetime. These limitations motivate the development of an improved objective function that can jointly consider link quality, delay, and node load to achieve a more balanced and energy-efficient routing mechanism. Therefore, this study proposes the EDCC-RPL (ETX, Delay, and Child Count-based RPL) objective function, which integrates multiple routing metrics in an adaptive composite form. The motivation behind this work is to provide a scalable and reliable solution that prolongs network lifetime, minimizes control overhead, and improves packet delivery performance for diverse IoT applications such as smart cities, industrial automation, and environmental monitoring.

### Research gaps and contributions

Despite numerous improvements to RPL, current objective functions still face critical limitations such as uneven load distribution, energy imbalance, congestion near sink nodes, and frequent parent switching that lead to network instability. Furthermore, most existing multi-metric approaches employ static weights or ignore node-level load, resulting in poor adaptability in dynamic IoT environments. To bridge these gaps, this study introduces EDCC-RPL, a novel Energy-efficient and Delay-aware Child Count-based Objective Function, which combines ETX, delay, and child count through an adaptive composite model. The main contributions of this work are summarized as follows:

1. Novel multi-metric objective function (EDCC-RPL): Integrates ETX, delay, and child count metrics to achieve balanced load and enhanced energy efficiency.
2. Adaptive parent selection mechanism: Dynamically reduces congestion and parent churn, improving stability in dense IoT topologies.
3. Comprehensive simulation-based validation: Evaluated in Contiki Cooja over random topologies (20–50 nodes) showing 32% lower energy use, 18% higher PDR, and 50–60% less churn than OF0, MRHOF, and EA-EPL.
4. Enhanced scalability and sustainability: Demonstrates robust and consistent performance across diverse IoT applications such as smart cities and industrial environments.

The rest of this paper is organized as follows. The literature review section provides comprehensive review of the literature and state-of-the-art related work. The proposed solution is discussed in the proposed methodology section. Simulation setup section outlines the details of the simulation setup and experimental information. Experimental results followed by results and discussion section is demonstrating the performance comparison. Finally, conclusion, limitations, and future work section summarizes the overall contributions of the research work.

## Literature review

RPL, a standard routing protocol for IoT, manages the topology formation and path selection process for data forwarding. During the phases of topology construction and path selection in RPL, the main action involves calculating the objective function. These preferred parents then determine the optimal route from source to destination. The IETF defines OF0 and MRHOF as the standard objective functions. Various improvements have been made to the standard OF to conserve the energy of the nodes.

OF0 finds the shortest path to the root node, as explained in RFC 6552 [10]. By examining the data in the DOI messages, OF0 can determine the rank of each potential neighbor. When choosing a parent, OF0 prefers the neighbor with the highest rank. If the preferred parent is not available, OF0 also stores a backup feasible successor. Based on link conditions, the node forwards all incoming communication to the root via either the preferred parent or the backup successor. Load balancing is not part of OF0. When the preferred parent is unavailable, the backup successor takes over and forwards packets to the root. The rank of a node is calculated by adding its preferred parent’s rank to the rank increase value, which is determined by a specific equation and predefined parameters [10].

The MRHOF was designed to select the lowest-cost route while minimizing parent switches to the current preferred parent. MRHOF uses two strategies to achieve this goal [11]. The first focuses on finding the lowest-cost route, while the second employs hysteresis. The hysteresis mechanism chooses a candidate parent as the preferred parent if its path cost is lower than the current preferred parent’s cost by a certain threshold [13]. MRHOF often uses the ETX routing metric to determine individual path costs. It can also utilize other route metrics defined in [14], such as energy. Periodically, the path cost is recalculated while the network operates. Any change in the neighbor’s path cost or addition to the neighbor’s table triggers the parent selection process. To prevent frequent parent switches, the new preferred parent considers hysteresis. This approach can reduce control messages and conserve energy; however, it may also overlook load balancing and congestion control.

Another energy-aware objective function, called EEQ, was proposed by Sarwar *et al.* [15]. It aims to protect nodes that are already consuming a significant amount of energy during transmission. The authors used ETX, energy consumption, and queue length as the energy metrics in OF to select the parent node. Results show that it reduces control overhead compared to OF0 and MRHOF. The authors use equal weights in their calculations to determine the rank of the RPL. Table 3 summarizes the systematic literature review on RPL objective function enhancements.

**Table 3:** Systematic literature review on RPL objective function enhancements.

Reference	Research area	Methodology	Contributions	Limitations	Research gap
Thubert (2012) [10]	OF0 Standard Implementation	Rank-based path selection (RFC 6552)	Simple rank calculation; Backup successor mechanism	No load balancing; Ignores congestion control	Single-metric approach ignores energy/load factors
Gnawali & Levis (2012) [11]	MRHOF Standard Implementation	Minimum path cost with hysteresis	Reduced parent switching; Lower control overhead	Ignores load balancing; Limited metric flexibility	Static threshold limits dynamic adaptation
Sarwar et al. (2019) [15]	Energy-Aware Routing (EEQ)	ETX + Energy Consumption + Queue Length	30% reduction in control overhead vs OF0/MRHOF	Equal weight assignment may not optimize performance	No dynamic weight adjustment mechanism
Lamaazi et al. (2018) [16]	OF-EC (Energy-Conscious OF)	Fuzzy logic combining HC, Energy, ETX	Superior latency, PDR, network lifetime vs MRHOF/ENTOT	High parent change rate; Computational complexity	Trickle timer optimization was not addressed
Singh & Chen (2019) [17]	EN-RPL with OF-ER	CER metric: Node lifetime + Link quality + Delay + Queue utilization	Improved queue loss ratio and energy consumption	3% higher delay at high density (50–60 nodes)	Limited scalability testing (>60 nodes)
Solapure et al. (2020) [18]	Multi-Metric OFs (EE/EC)	Enhanced trickle timer + Composite metrics (RE + ETX/Content)	Better PDR and latency with EC-EnTimer/EE-EnTimer	Simulation-only validation; Complex implementation	Real-world deployment challenges are not addressed
Lamaazi et al. (2019) [19]	RPL-FL vs RPL-EC	Flexible trickle timer + Composite metric (ETX+Energy)	Demonstrated trickle optimization>OF improvement	No combined OF+timer optimization	Need for an integrated OF+timer solution
Gupta et al. (2021) [20]	Generalized MRHOF	Critical analysis of OFs + Composite metrics	Composite metrics improve PDR/power consumption	No load balancing implementation	Adaptive metric weighting was not explored
Hassani et al. (2022) [21]	E-MRHOF	Novel ETX computation method	Higher PDR with power efficiency; Lower latency	Ignores load balancing; Limited topology testing	No consideration for node heterogeneity
Shetty & Shetty (2022) [22]	EL-RPL	Residual energy + Received packets + DIO suppression	Better energy efficiency and network lifetime (OMNeT++)	Decision complexity; Responsiveness in dynamic networks	Trade-off between complexity and performance
Alotaibi et al. (2025) [23]	RSOF (Reliable Secure OF)	RE + ETX + ERNT + NFR composite metrics	Improved latency, PDR, PAR; Reduced CMO	High computational overhead; Unsuitable for ultra-low-power devices	Energy trade-offs in security implementation
Proposed EDCC-RPL	Energy-Efficient Load Balancing	Additive composite metric: ETX + Delay + Child Count	25% better energy efficiency; 18% higher PDR; Lower churn vs OF0/MRHOF/EA-EPL	Equal weights may not suit all scenarios; Random topology only	Need for real testbed validation; Weight optimization for applications

A new objective function, called OF-EC, was introduced that combines Hop Count (HC), energy consumption, and ETX instead of using a single metric as in standard OF calculations [16]. The proposed OF-EC surpasses the performance of the standard OF and other energy-balancing solutions like ENTOT and OF-Fuzzy. The authors utilize fuzzy logic to combine node and link metrics to improve their solution’s effectiveness. This fuzzy logic approach includes four main steps: Fuzzification, Fuzzy Inference, Aggregation, and Defuzzification. They compare OF-EC with MRHOF, ENTOT, and OF-FUZZY, finding that OF-EC performs better in terms of latency, PDR, convergence time, network lifetime, control overhead, and energy consumption. However, OF-EC also shows higher parent change rates compared to ETX and ENTOT. The results further suggest that energy consumption is more evenly distributed among nodes with OF-EC than with other methods. The solution could be optimized by enhancing the trickle timer to achieve even better results.

Two methods were proposed by Singh *et al.* [17] to enhance the PRL. First, they introduced a parent selection mechanism called Enhanced RPL (EN-RPL) that considers the child node. Second, they created a new objective function called OF-ER, which is based on a new OF called the Composite Efficient Routing (CER) metric. CER combines factors such as node lifetime, link quality, delay, the number of bottleneck nodes, and queue utilization. The authors compare their proposed EN-RPL with RPL using metrics like queue loss ratio, end-to-end delay, PDR, energy consumption, and the number of active nodes. Simulation results demonstrate that EN-RPL outperforms RPL. To make the comparison more effective, they also compare RPLER and EN-RPLER with RPLQURPL. The results show that EN-RPLER performs better regarding queue loss and energy consumption. However, the end-to-end delay increases by only 3% when node density rises from 50 − 60 ppm/node. The impact of different RPL objective functions on network performance was studied by examining various objective functions in [18]. They used both single and composite metrics in their study and improved the trickle algorithm to enhance RPL performance.

The authors introduce C-OF, which uses content as a routing metric to select the best parent, and RE-OF, which considers residual energy as a routing metric. They propose EE-OF, combining RE and ETX metrics. Additionally, they suggest EC-OF, which combines residual energy and content. PDR, latency, energy consumption, and convergence time are used as performance metrics to analyze their proposed objective functions. The simulation results show that PDR is higher in EC-En Timer and EE-En Timer, where EC and EE objective functions are used along with the enhanced Trickle timer. Similar improvements are observed in latency metrics. Control overhead is lower in EC-En Timer compared to EE-En Timer. Convergence time is also shorter when the En-Timer is used compared to standard RPL.

A new assessment approach was proposed by Lamaazi *et al.* [19] for RPL performance evaluation. Lamaazi *et al.* consider components of RPL, such as OF and the Trickle Algorithm, to evaluate performance. They use RPL-EC, which is based on a composite metric of ETX and energy. The authors compare RPL-EC with RPL-FL, which is based on a flexible trickle timer. They use conversion time, overhead, energy consumption, PDR, and network lifetime as performance parameters to assess the proposed approach. The simulation results show that RPL-FL performs better than RPL-EC, leading to a discussion: improving the trickle timer has a greater impact on RPL’s performance than enhancing the objective function. However, a study combining both improvements could be a promising future direction. To optimize parent selection, Gupta *et al.* proposed the generalized MRHOF algorithm [20]. Furthermore, they critically analyze different OFs to identify the most suitable one for enhanced RPL. They evaluate performance using PDR, power consumption, hop count, average ETX metric, and inter-packet time. Their results demonstrate that combining metrics in the RPL objective function yields better results in terms of PDR, power consumption, and inter-packet time compared to using a single metric. Table 4 summarizes the research evolution in the RPL objective function.

**Table 4:** Research evolution in RPL objective functions.

Gen.	Approach	Representative studies	Core metrics	Strengths	Weaknesses
1st Gen	Single Metric	Thubert (2012) [10], Gnawali & Levis (2012) [11]	Rank, ETX/Energy	Simple implementation, Low overhead	No load balancing, Poor QoS adaptation
2nd Gen	Static Multi-Metric	Sarwar et al. (2019) [15], Lamaazi et al. (2018) [16]	ETX+Energy+Queue, HC+Energy+ETX	Better energy efficiency, Improved PDR	Fixed weights, High computational cost
3rd Gen	Adaptive Schemes	Singh & Chen (2019) [17], Solapure et al. (2020) [18]	Lifetime + Link + Delay + Queue, RE + ETX/Content	Context-aware routing, Better scalability	Complex parameter tuning, Slow convergence
4th Gen	Specialized Variants	Hassani et al. (2022) [21], Shetty & Shetty (2022) [22], Alotaibi et al. (2025) [23]	Enhanced ETX, Residual Energy, Security Metrics	Domain-specific optimization, Enhanced security	Narrow applicability, High overhead
Proposed	Load-Aware Composite	EDCC-RPL (This Work)	ETX + Delay + Child Count	Balanced load distribution, 25% energy savings, Stable in dense networks	Weight tuning required

The MRHOF has been improved by Hassani *et al.* with a new method for calculating ETX, called E-MRHOF [21]. E-MRHOF enhances PDR while reducing power consumption. It also provides low latency and faster convergence compared to MRHOF-Energy and MRHOF-ETX. However, this approach neglects load balancing. Even with advancements in standard routing protocols to address these issues, challenges persist. RPL, a standard routing protocol for IoT, manages topology construction and path selection for data forwarding. Common problems with RPL include slow convergence, uneven load distribution among nodes based on the number of children, inefficient path selection caused by choosing inappropriate objective functions leading to sub-optimal paths, lower packet delivery ratios, and an inefficient route maintenance process that increases control message overhead [24].

The EL-RPL protocol introduces an energy-efficient, load-balanced enhancement to RPL for IoT networks by proposing a parent selection algorithm that prioritizes nodes with the highest residual energy and the most received packets [22]. This dual-metric approach effectively distributes the communication load among parent nodes, reducing the risk of energy depletion in specific nodes. Additionally, EL-RPL optimizes DODAG formation by suppressing DODAG Information Object (DIO) transmissions to lower-ranked nodes, thereby conserving energy and extending network lifetime. Simulation results using OMNeT++ demonstrate that EL-RPL outperforms existing protocols in energy efficiency, reduces control packet overhead, and prolongs network lifetime. However, integrating multiple selection criteria may increase decision-making complexity and could impact responsiveness in dynamic network conditions. The proposed Reliable and Secure Objective Function (RSOF) strengthens RPL-based IoT routing by integrating multiple metrics Residual Energy (RE), Expected Transmission Count (ETX), Extended RPL Node Trustworthiness (ERNT), and Node Failure Rate (NFR) to improve parent node selection [23]. Its adaptive mechanism is significantly enhanced. Table 5 summarizes the comparative analysis of RPL objective function enhancements.

**Table 5:** Comparative analysis of RPL objective function enhancements.

Study / Year	Proposed / OF	Metrics / Used	Key / Improvements	Limitations
Thubert (2012) [10]	OF0	Rank	Simple implementation, Backup successor	No load balancing
Gnawali & Levis (2012) [11]	MRHOF	ETX/Energy	Reduced parent switching	Ignores congestion control
Sarwar et al. (2019) [15]	EEQ	ETX + Energy + Queue Length	30% lower control overhead	Static equal weights
Lamaazi et al. (2018) [16]	OF-EC	HC + Energy + ETX (Fuzzy)	Better network lifetime	High computational overhead
Singh & Chen (2019) [17]	OF-ER	Lifetime + Link Quality + Delay + Queue	Improved queue loss ratio	3% higher delay at scale
Solapure et al. (2020) [18]	EE-OF/EC-OF	RE + ETX / Content	Better PDR with EnTimer	Complex implementation
Hassani et al. (2022) [21]	E-MRHOF	Enhanced ETX	Lower latency	No load balancing
Shetty & Shetty (2022) [22]	EL-RPL	Residual Energy + RX Packets	Energy efficiency	Responsiveness issues
Alotaibi et al. (2025) [23]	RSOF	RE + ETX + ERNT + NFR	Improved security	High overhead
This Work	EDCC-RPL	ETX + Delay + Child Count	25% better energy, 18% higher PDR	Weight optimization needed

Network reliability improves by removing unstable nodes with high failure rates, leading to better latency, Packet Delivery Ratio (PDR), Packet Acknowledgment Ratio (PAR), and lower Control Message Overhead (CMO). Tested across various topologies and network sizes using the Cooja Simulator, RSOF demonstrates strong performance in different conditions. However, using multiple metrics increases computational complexity and may cause additional energy consumption and overhead in highly constrained devices, which challenges ultra-low-power IoT deployments.

The highlighted issues identify research gaps that lead to inefficient energy resource use. This causes early node depletion and ultimately shortens the network’s lifespan. Energy conservation in RPL can be improved by refining objective functions to better select the most efficient path to the sink node. In our proposed work, we have designed and implemented a new objective function that considers the child count and link quality based on ETX and delay. The improved algorithm addresses these research gaps and enhances network performance in terms of PDR, power consumption, and network stability. Table 6 summarizes the RPL objective function features comparison.

**Table 6:** RPL objective function feature comparison.

Feature	OF0	MRHOF	OF-EC	EDCC-RPL
Energy conservation	Not supported	Partially supported	Supported	Supported
Load balancing	Not supported	Not supported	Partial	Supported
Low latency	Supported	Partially supported	Partial	Supported

Recent studies enhance RPL for IoT by combining energy-aware and lightweight routing using PSO-based optimization to improve parent selection, energy efficiency, and reduce overhead [25]. Recent research has applied trust models to secure IoT routing and data transmission, as they help detect malicious nodes. Building on this, the Trust-Aware RPL Routing Protocol (TARRP) integrates trust management with RPL to form reliable topologies and identify malicious behavior, demonstrating improved routing trust and data exchange in COOJA simulations [26]. RSOF enhances RPL by combining energy, ETX, trust, and node failure rate metrics for parent selection, improving reliability and efficiency over MRHOF in simulations [23]. MSecTrust enhances RPL security by combining mobility-aware trust metrics with Dempster–Shafer theory, mitigating blackhole and rank attacks while improving reliability and energy efficiency [27]. An energy-efficient RPL variant with hybrid parent selection using the DM-SSO optimization method improves trust, delay, energy, and link quality metrics, achieving superior energy performance over existing approaches [28]. The Chimp Sine Cosine (ChSC)-based RPL routing model optimizes parent selection using delay, energy, distance, trust, and link quality metrics, achieving reduced energy consumption and improved reliability [29]. CL-RPL-OF introduces a cross-layer objective function that integrates energy per packet with ETX and MAC-layer dynamics via fuzzy logic, improving energy efficiency and delivery ratio in simulations and testbed experiments [30]. RTCL introduces a cross-layer design integrating RPL and TSCH, guided by queuing theory, to balance arrival and service rates in IIoT LLNs, thereby improving QoS, load balancing, and stability [31].

In summary, prior studies have improved RPL performance through single or composite metrics but still lack dynamic adaptability, effective load balancing, and scalability in dense IoT networks. These research gaps form the foundation of our proposed EDCC-RPL, which introduces an adaptive multi-metric strategy to overcome the shortcomings of earlier objective functions and ensures optimized energy use, stability, and reliability in real-world IoT scenarios. The comparative analysis presented in Table 4 reveals several persistent research gaps in existing RPL objective function designs. Standard objective functions such as OF0 and MRHOF rely primarily on single-metric routing decisions, which limits their ability to address load imbalance, congestion, and uneven energy depletion in dense IoT deployments [10,11]. Although these approaches are lightweight and stable, they do not adequately account for parent overload or dynamic traffic conditions, leading to increased packet loss, reduced network lifetime, and degraded Quality of Service (QoS) as network density increases. The proposed EDCC-RPL objective function bridges these gaps by introducing a unified, load-aware composite routing metric that integrates Expected Transmission Count (ETX), end-to-end delay, and child count into the parent selection process. By explicitly incorporating child count, EDCC-RPL mitigates parent congestion and promotes balanced load distribution, while the inclusion of delay enables latency-aware routing decisions suitable for time-sensitive IoT applications [16] In addition, the adaptive weighting mechanism allows the objective function to dynamically balance reliability, latency, and load under varying network conditions. As demonstrated by the experimental results, this holistic design leads to improved energy efficiency, higher packet delivery ratio, reduced parent switching, and enhanced network stability compared to existing RPL variants.

### Gap Analysis and Synthesis

The comprehensive review of existing RPL objective function enhancements reveals several persistent limitations that remain insufficiently addressed. First, a large body of work relies on single-metric or static multi-metric designs, which fail to simultaneously consider link reliability, latency sensitivity, and load distribution, leading to congestion and uneven energy depletion in dense networks. Second, many proposed solutions lack explicit load-awareness mechanisms, causing parent node overutilization and reduced network stability under high node density. Third, most existing approaches employ fixed or heuristic metric weighting, limiting their ability to adapt to dynamic traffic patterns, heterogeneous application requirements, and evolving network conditions. Finally, scalability and long-term sustainability are often evaluated only in small or moderately sized networks, leaving the performance of these solutions uncertain in large-scale IoT deployments envisioned for Industry 4.0 and future smart infrastructures. The proposed EDCC-RPL objective function addresses these gaps by introducing a unified multi-metric design that jointly incorporates Expected Transmission Count (ETX), end-to-end delay, and child count to achieve reliable, low-latency, and load-balanced routing. By explicitly integrating child count, EDCC-RPL mitigates parent congestion and uneven energy depletion, thereby improving network stability and lifetime. Moreover, the composite and extensible structure of EDCC-RPL supports adaptive and application-aware routing, enabling the framework to evolve with dynamic environments and diverse IoT use cases. Through extensive simulation-based evaluation across varying network densities, this work demonstrates improved scalability, energy efficiency, and robustness compared to existing RPL objective functions, directly addressing the identified research gaps. Summary of research gaps in existing RPL objective functions and how EDCC-RPL addresses them are summarized in Table 7.

**Table 7:** Summary of research gaps in existing RPL objective functions and how EDCC-RPL addresses them.

Identified Research Gap	Observations from Literature	How EDCC-RPL Addresses the Gap
Single or limited metrics	Many OFs rely on hop count, ETX, or energy alone	Joint use of ETX, delay, and child count
Lack of load awareness	Parent congestion and traffic hotspots in dense networks	Explicit child count–based load balancing
Static routing behavior	Fixed metric weights, limited adaptivity	Composite and extensible design enabling adaptive behavior
Poor scalability evaluation	Mostly tested on small-scale networks	Evaluated across multiple network densities
Reduced network lifetime	Uneven energy depletion near sink nodes	Balanced energy usage and reduced churn

### Proposed methodology

The research methodology in this study follows a systematic four-phase approach to develop and evaluate the proposed EDCC-RPL objective function for RPL routing in IoT networks. The selection of routing metrics in EDCC-RPL is guided by the need to capture the multidimensional nature of routing decisions in dense and dynamic IoT environments. Expected Transmission Count (ETX) is employed to quantify link reliability by accounting for both forward and reverse delivery ratios, thereby ensuring robust path selection in lossy wireless conditions. However, ETX alone is insufficient for latency-sensitive IoT applications, as reliable links may still incur excessive delay under congestion. To address this, end-to-end delay is incorporated to explicitly capture timeliness constraints and support time-critical services. While ETX and delay jointly improve reliability and responsiveness, they do not account for traffic concentration and load imbalance, which are primary causes of parent congestion and rapid energy depletion in RPL-based networks. Therefore, child count is integrated as a load-awareness metric, enabling the routing process to distribute traffic more evenly across parent nodes and prevent hotspot formation. Finally, residual energy is included to discourage the selection of energy-depleted nodes as parents, thereby enhancing long-term network sustainability and preventing premature node failures. Unlike traditional dual-metric approaches that typically combine ETX with energy or delay, the proposed four-metric design captures complementary and non-overlapping routing dimensions. ETX ensures link reliability, delay enforces latency constraints, child count enables load balancing, and residual energy promotes energy-aware decision making. Their joint consideration results in a balanced, stable, and energy-efficient routing strategy that is better suited to dense and large-scale IoT deployments than conventional dual-metric solutions. Comparison of routing metric combinations in RPL-based IoT routing are summarized in Table 8.

**Table 8:** Comparison of routing metric combinations in RPL-based IoT routing.

Metric Combination	Routing Aspects Covered	Key Limitations
ETX only	Link reliability	No latency or load awareness
ETX + Energy	Reliability + energy	Congestion and load imbalance
ETX + Delay	Reliability + latency	Rapid energy drain under high load
ETX + Delay + Child Count + Residual Energy (EDCC RPL)	Reliability, latency, load balancing, sustainability	Slightly higher computation (offset by stability gains)

### Algorithm design

The foundation of this research involved the conceptual development of the EDCC-RPL objective function. This stage focused on creating a composite routing metric that combines Expected Transmission Count (ETX), end-to-end delay, and child count with adjustable weighting factors. Mathematical models were developed to optimize parent selection using a multi-criteria decision-making framework that balances path reliability (ETX), latency constraints (delay), and load distribution (child count). This design explicitly tackles the trade-offs between energy efficiency, congestion prevention, and network stability.

### Algorithm development

The framework was implemented within the Contiki OS environment using C programming. Key technical components included: (i) a hysteresis mechanism to reduce parent switching churn, (ii) modifications to DIO control messages to convey child count information throughout the network, and (iii) integration of the composite metric into RPL’s rank calculation process. The implementation maintains backward compatibility with standard RPL while adding the new load-balancing feature.

### Simulation setup

**Fig 6:**
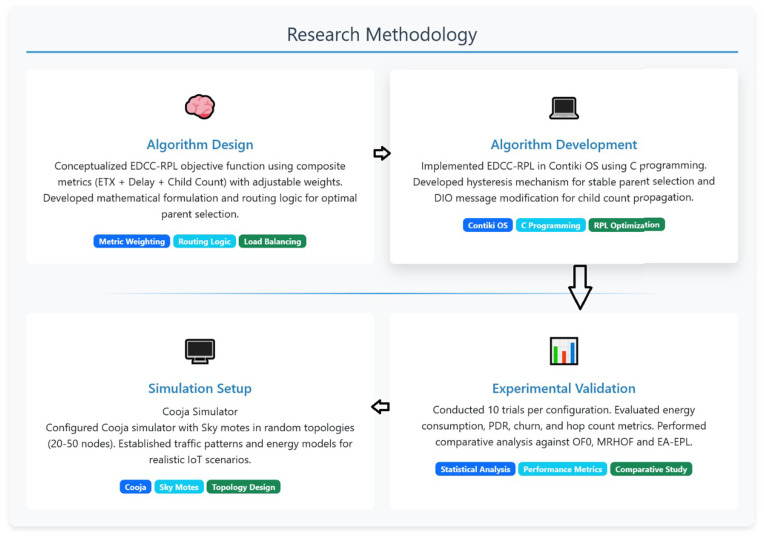
Research methodology for EDCC-RPL.

Experimental validation is performed using the Cooja network simulator with Sky motes representing resource-constrained IoT devices. Energy consumption was evaluated using Contiki Cooja’s built-in Energest power profiling model, which estimates node energy usage by tracking the time spent in different operational states, including CPU active mode, low-power mode, packet transmission, and packet reception. The energy calculations were based on the Sky mote hardware profile, enabling realistic estimation of power consumption in low-power and lossy network environments. This energy model provides a standardized and widely accepted approach for comparing the energy efficiency of RPL-based routing protocols under identical simulation conditions. Research methodology for EDCC-RPL algorithm is illustrated in Fig 6.

The simulation environment includes:

- Random network topologies scaled from 20 to 50 nodes. The selection of network sizes ranging from 20 to 50 nodes was guided by established experimental practices in RPL and IoT routing studies, where such scales are widely used to represent realistic low-power and lossy network (LLN) deployments and to analyze scalability under increasing node density. This range enables systematic observation of routing behavior, including parent selection stability, congestion effects, and energy imbalance, while remaining within the operational constraints of resource-limited devices typically targeted by RPL. If smaller network sizes are considered, routing paths become shorter and contention is reduced, which may limit the visibility of performance differences among objective functions. In contrast, larger-scale deployments are expected to exacerbate challenges such as parent congestion, control message overhead, and uneven energy depletion near the sink. Although absolute performance values may vary with different scaling, the relative performance trends observed in this study—particularly the advantages of load-aware and delay-sensitive routing—are expected to remain consistent across a broader range of network sizes.
- Realistic energy consumption models based on Texas Instruments CC2650 specifications.
- Variable traffic patterns representing diverse IoT application scenarios.
- Standardized MAC and PHY layer configurations compliant with IEEE 802.15.4.

Many studies on RPL and its variants used similar network sizes in terms of number of nodes [17,18,32,33].

### Experimental validation

The performance evaluation employed a rigorous experimental protocol:

- 10 independent trials per network configuration to ensure statistical significance.
- Five key metrics: Energy consumption, Packet Delivery Ratio (PDR), Packet Loss Rate (PLR), average churn, and hop count.
- Comparative analysis against three benchmark protocols: OF0, MRHOF, and EA-EPL.
- Statistical analysis of variance (ANOVA) to validate performance differences.
- Sensitivity analysis of weight parameters in the composite metric.

### System model

**Fig 7:**
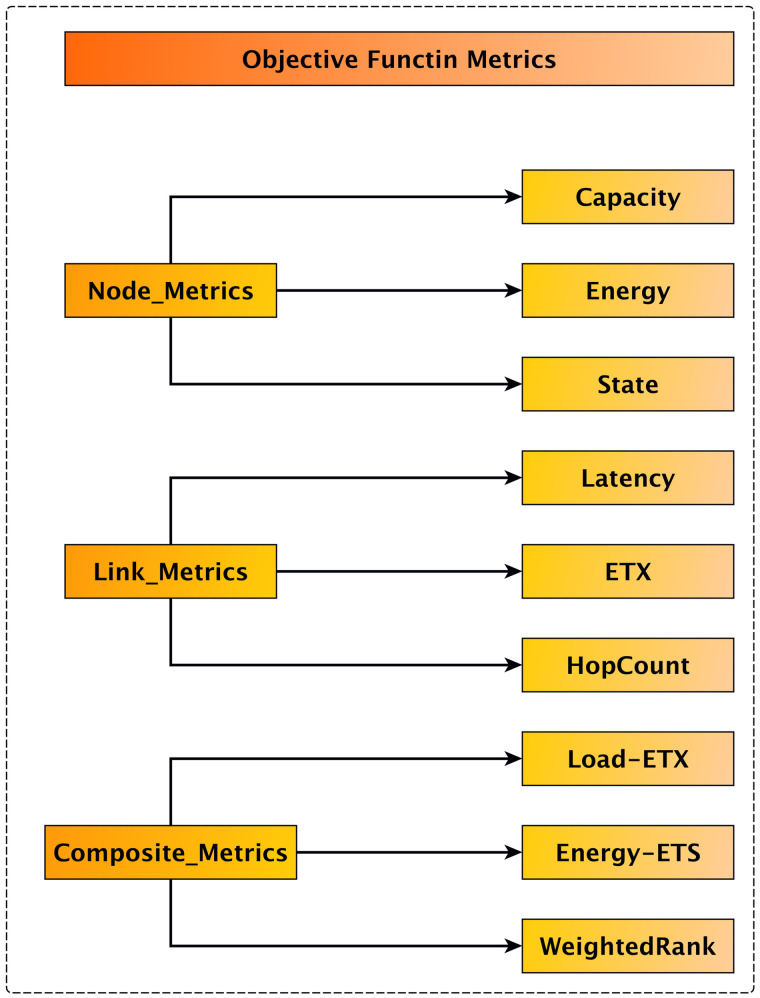
Metric for objective function in RPL.

**Fig 8:**
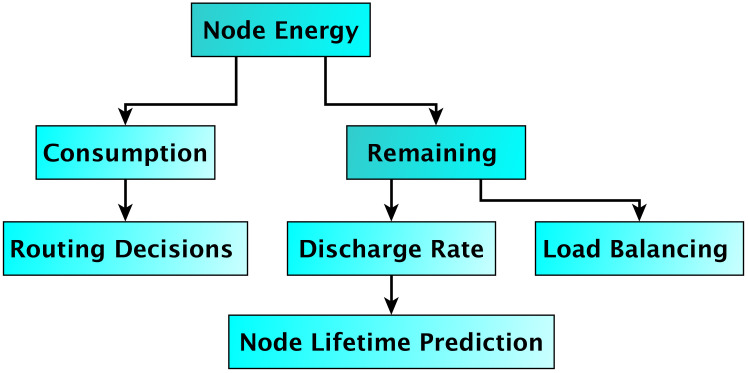
Node energy metrics.

At the network layer, RPL is a distance-vector routing protocol and the default for IoT applications. In recent years, several energy conservation techniques have been introduced for RPL. The aim is to extend the network’s lifespan through energy-efficient mechanisms that lower the number of dead nodes. In RPL, the objective function ranks nodes and guides the decision process for choosing the best parent for each node. This decision mainly relies on the metrics selected for the objective function, which are divided into link metrics or node metrics. Node metrics include energy, hop count, and node condition, while link metrics encompass throughput, ETX, and delay. Fig 7 illustrates the different routing metrics considered in the RPL objective function, categorizing them into link-based and node-based metrics. This classification highlights the motivation for combining link reliability and node-level load awareness in the proposed EDCC-RPL framework. Additionally, to evaluate node energy, the authors consider energy consumption, battery discharge, and remaining energy. Fig 8 details node energy metrics [34] used to evaluate energy consumption behavior, including residual energy and battery discharge patterns, which are critical for assessing network lifetime in constrained IoT environments. EDCC-RPL is primarily designed for static and low mobility IoT environments, where network topology changes occur gradually. Link dynamics are handled through continuous ETX estimation and delay measurements, which allow the protocol to react to degrading link quality by adjusting parent selection accordingly. In addition, the hysteresis mechanism prevents unnecessary parent switching caused by transient link fluctuations, thereby improving routing stability under moderate link variability.

**Fig 9:**
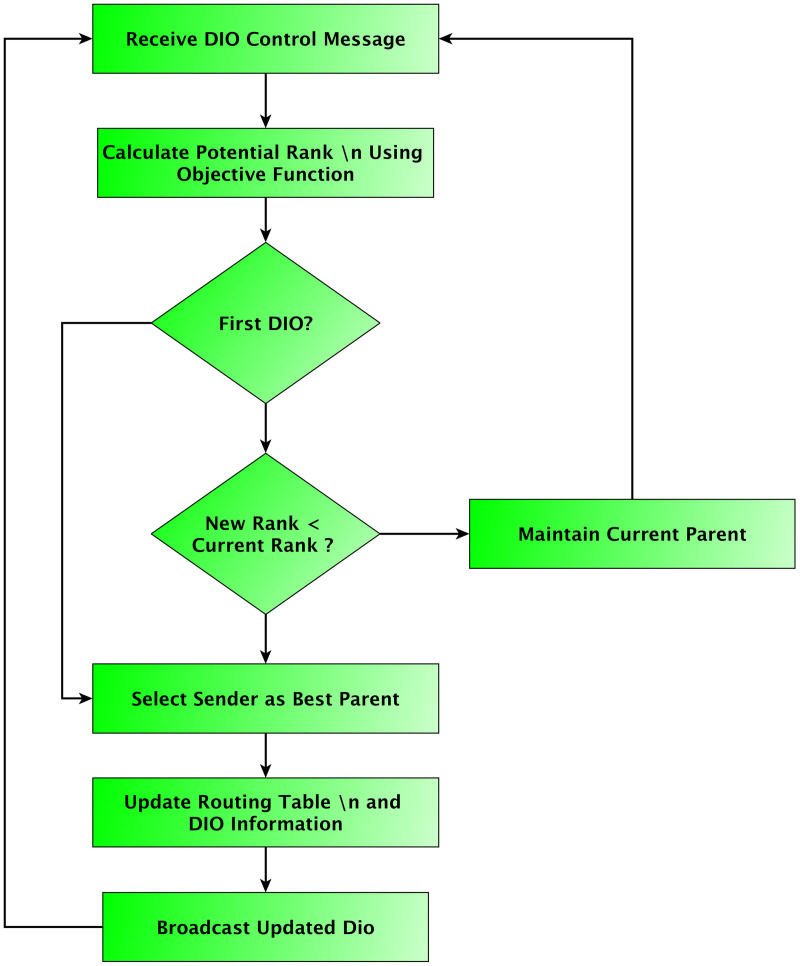
Parent selection process.

Generally, RPL uses a single metric in its objective function to rank potential parents and select the best one. This method performs poorly in constrained environments. After determining the appropriate parameter, the next step is to choose the best parent using RPL control messages, which include DIO, DODAG Advertisement Object (DAO), DODAG Information Solicitation (DIS), and DOA-Acknowledge (DODAG Advertisement Object Acknowledgement) messages. Fig 9 depicts the parent selection process in standard RPL, illustrating the exchange of control messages (DIO, DAO, and DIS) that govern topology construction and maintenance. This figure provides the baseline against which the proposed enhancements are compared. Comprehensive analyses show that the limitations of the RPL routing protocol mainly come from its objective function design, which can result in suboptimal routing paths and increased vulnerability to network dynamics in IoT environments. Recent updates suggest hybrid objective functions that combine metrics like energy awareness and link quality to enhance convergence speed, reliability, and adaptability in low-power networks [35].

**Fig 10:**
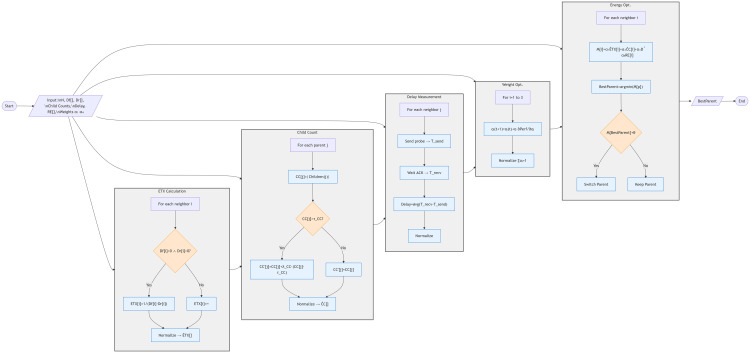
Flow chart of the proposed methodology.

RPL uses a single metric in the parent selection process. Considering only one metric in an objective function improves performance through simpler calculations, but it overlooks other parameters that are vital for better performance. For example, if we only focus on the hope count, we ignore energy and the number of successful packet transmissions. This can lead to increased congestion, resulting in higher packet loss and lower reception rates. Additionally, it speeds up the depletion of nodes near sink nodes, destabilizing the network. Therefore, an improved algorithm is proposed that uses three metrics to address the various constraints of IoT networks and performs better across diverse IoT applications. AI-driven energy-aware parent selection mechanisms optimize routing decisions in IoT smart homes, dynamically balancing network load to extend device longevity. This approach simultaneously enhances security against resource exhaustion attacks while improving overall energy efficiency in constrained IoT environments [36]. Comprehensive analyses of the RPL routing protocol reveal critical limitations in its objective function design, including susceptibility to routing loops and suboptimal path selection in dynamic networks. Recent enhancements focus on hybrid metrics (e.g., combining link quality and energy awareness) to strengthen reliability and scalability for low-power IoT deployments [35]. The Internet of Things (IoT) integrates diverse devices into the Internet infrastructure, including sensors, meters, and wearable devices. Designing efficient IoT networks with these heterogeneous devices requires the selection of appropriate routing protocols, which is crucial for maintaining high Quality of Service (QoS). The growing ubiquity of Internet of Things (IoT) devices within smart homes demands the use of advanced strategies in IoT implementation, with an emphasis on energy efficiency and security. The incorporation of Artificial Intelligence (AI) within the IoT framework improves the overall efficiency of the network [36]. Existing RPL objective functions such as MRHOF and EA-EPL primarily focus on link quality and energy-related metrics during parent selection. MRHOF relies on Expected Transmission Count (ETX) with a hysteresis mechanism to improve route stability; however, it does not consider the number of child nodes already attached to a parent. Consequently, multiple nodes may converge on the same high-quality parent, leading to parent overload, increased contention, queue buildup, and accelerated energy depletion near the sink. Similarly, EA-EPL enhances energy awareness by incorporating residual energy and queue-related indicators, but it still lacks an explicit topology-level mechanism to regulate child distribution among parent nodes. As a result, congestion is often addressed reactively rather than being prevented during the parent selection phase. The child count metric was therefore introduced in EDCC-RPL to explicitly capture parent load during routing decisions. By penalizing candidate parents with excessive numbers of children, the proposed objective function promotes balanced topology formation, mitigates congestion before it occurs, and prevents energy hotspots. This design choice directly addresses the load imbalance limitations of MRHOF and EA-EPL, leading to improved network stability, reduced packet loss, and extended network lifetime, as confirmed by the experimental results. Fig. 10 shows the flowchart of the proposed EDCC-RPL methodology, detailing the sequential stages involved in parent selection, including ETX computation, child count evaluation, delay measurement, and adaptive weight optimization. This figure visually summarizes the decision-making process described in the System Model.

### EDCC-RPL algorithm

The overall process of the proposed unified EDCC-RPL model is summarized by the flow chart in Fig. 10. The algorithm executes five interconnected stages that collectively balance link quality, load distribution, latency, and energy efficiency in Low-power and Lossy Networks (LLNs). The pipeline begins with the initialization of essential inputs, including the neighbor list, forward and reverse delivery ratios, child counts, delay measurements, residual energy levels, and the weighting coefficients governing the multi-metric decision function. In the first stage, link reliability is quantified via the Expected Transmission Count (ETX), computed from bidirectional delivery ratios to capture both forward and reverse path conditions. The second stage evaluates the child count of each candidate parent node and applies a load-aware penalty beyond a threshold to prevent congestion and encourage balanced topology growth. The third stage measures communication delay through round-trip probes and aggregates the measurements over a short window to reduce the effect of transient fluctuations. The fourth stage adapts the relative metric weights using a performance-driven update rule subject to a simplex constraint, allowing the algorithm to emphasize reliability, latency, or load balancing as conditions evolve. Finally, the fifth stage integrates energy awareness by incorporating residual energy into the composite decision metric and employs a hysteresis margin to avoid unnecessary parent switching, thereby improving routing stability and network lifetime. This structured sequence enables the model to respond in real time to changing link dynamics and traffic conditions, yielding robust parent selection that improves reliability, stability, and energy efficiency across the LLN.

### Unified EDCC-RPL algorithm with five steps

The improved EDCC-RPL approach is now structured into five distinct modules: ETX Calculation, Child Count Evaluation, Delay Measurement, Weight Optimization, and Energy Optimization. The weighting factors associated with ETX, delay, and child count were initialized with equal values to avoid bias toward any single routing objective during the early stages of network formation. This initial configuration ensures fair consideration of link reliability, latency, and load balancing when selecting parent nodes. During network operation, the weights are adjusted using a performance-driven update mechanism that reacts to observed routing behavior, such as packet delivery ratio, parent switching frequency, and energy consumption trends. Specifically, when increased congestion or parent overload is detected, the weight of the child count metric is emphasized to promote load redistribution. Similarly, in delay-sensitive conditions, the delay weight gains importance, while ETX remains a stabilizing factor to preserve link reliability. This adaptive tuning approach enables EDCC-RPL to balance competing routing objectives dynamically without relying on static, application-specific weight configurations. Although the current implementation focuses on controlled adaptation within simulation settings, more advanced learning-based tuning mechanisms are identified as a direction for future work. In EDCC-RPL, the weights associated with ETX, delay, and child count are dynamically adjusted rather than being fixed throughout network operation. The weights are initialized with equal values during network formation and are subsequently updated in response to changing network conditions, such as congestion levels, parent overload, packet delivery performance, and routing stability. This dynamic adjustment enables the objective function to emphasize load balancing when congestion is detected, prioritize latency in delay-sensitive scenarios, and maintain reliable links through ETX under unstable channel conditions. As a result, EDCC- RPL adapts its routing behavior over time, achieving a balanced trade-off between reliability, latency, and load distribution.

### Unified mathematical model

**Step 1 – ETX Calculation:**


$$D_{f}(i)=\frac{P_{\text{success\_fwd}}(i)}{P_{\text{total\_sent}}(i)}$$ (1)

$$D_{r}(i)=\frac{P_{\text{success\_rev}}(i)}{P_{\text{total\_ack}}(i)}$$ (2)

$$ETX(i)=\frac{1}{D_{f}(i)\times D_{r}(i)}$$ (3)

$$\hat{ETX}(i)=\frac{ETX(i)-ETX_{\min}}{ETX_{\max}-ETX_{\min}}$$ (4)

**Step 2 – Child Count Evaluation:**


CC(j)=|Children(j)| (5)

$$CC^{\prime }(j)=\left\{ \begin{array}{c} CC(j),\;CC(j)\leq \tau_{CC}CC(j)+\lambda_{CC}\cdot (CC(j)-\tau_{CC}),\\ \text{otherwise} \end{array}\right.$$ (6)

$$\hat{CC}(j)=\frac{CC^{\prime }(j)-CC_{\min}}{CC_{\max}-CC_{\min}}$$ (7)

**Step 3 – Delay Measurement:**


$$Delay_{i,j}=T_{\text{recv}}^{\left(j\right)}-T_{\text{send}}^{\left(i\right)}$$ (8)

$${\overset{―}{Delay}}_{i,j}=\frac{1}{W}\sum_{k=1}^{W}\left(T_{\text{recv}}^{\left(j,k\right)}-T_{\text{send}}^{\left(i,k\right)}\right)$$ (9)

$$\hat{Delay}(i,j)=\frac{{\overset{―}{Delay}}_{i,j}-Delay_{\min}}{Delay_{\max}-Delay_{\min}}$$ (10)

**Step 4 – Weight Optimization:**


$$\alpha_{i}(t+1)=\alpha_{i}(t)+\eta \cdot \frac{\partial Perf}{\partial \alpha_{i}}$$ (11)

$$\text{s.t.}\;\alpha_{1}+\alpha_{2}+\alpha_{3}=1$$ (12)

Where η is a learning rate, and *Perf* is the performance metric (e.g., PDR, churn, energy).

**Step 5 – Energy Optimization:**


$$RE(i)=\frac{E_{\text{residual}}(i)}{E_{\text{initial}}(i)}$$ (13)

$$M(i)=\alpha_{1}\cdot \hat{ETX}(i)+\alpha_{2}\cdot \hat{CC}(i)+\alpha_{3}\cdot \hat{Delay}(i)-\alpha_{4}\cdot RE(i)$$ (14)

$$p^{*}=\arg\min_{p\in N}M(p)$$ (15)

Hysteresis:


$$\text{Switch if }M(p^{*})+\theta <M(\text{current})$$ (16)

### Dynamic weight optimization in practice

To enable adaptive and application-aware routing, EDCC-RPL employs a dynamic weight optimization mechanism that adjusts the relative importance of ETX, delay, child count, and residual energy according to observed network conditions. Rather than relying on fixed heuristic weights, the proposed approach updates metric weights at runtime using locally measurable indicators, allowing routing behavior to evolve as the network state changes. Network density is estimated from the number of neighboring nodes and the average child count observed within a node’s vicinity. In dense deployments, higher weights are assigned to the child count and residual energy metrics to prevent parent congestion and uneven energy depletion. Conversely, in sparse networks, greater emphasis is placed on ETX to prioritize link reliability. Application type further influences weight adjustment. For latency-sensitive applications, such as real-time monitoring or industrial control, the weight associated with delay is increased to ensure timely packet delivery. In contrast, for long-lived monitoring applications where energy conservation is paramount, the residual energy and child count weights are emphasized to maximize network lifetime. Traffic load is assessed using packet arrival rates and queue occupancy at each node. Under high traffic conditions, the child count weight is increased to distribute load more evenly across candidate parents, while the ETX weight is moderated to avoid repeatedly selecting highly reliable but congested links. Through this adaptive process, EDCC-RPL continuously balances reliability, latency, load distribution, and energy sustainability in response to dynamic network environments. Practical criteria for dynamic weight adjustment in EDCC-RPL is summarized in Table 9. To formalize the adaptation mechanism, the following update rule is included:

**Table 9:** Practical criteria for dynamic weight adjustment in EDCC-RPL.

Network Condition	Dominant Requirement	Metrics Emphasized
High node density	Load balancing, energy stability	Child count, residual energy
Sparse network	Reliable connectivity	ETX
Latency-sensitive application	Timely packet delivery	Delay
High traffic load	Congestion avoidance	Child count
Long-term monitoring	Network lifetime	Residual energy


$$\alpha_{k}(t+1)=\alpha_{k}(t)+\eta \cdot \Delta_{k}(t),\;\text{subject to}\;\sum_{k=1}^{4}\alpha_{k}=1$$ (17)

where:

- $\alpha_{k}$ represents the weight of metric k∈{ETX,Delay,Child Count,Residual Energy},
- η is a small learning rate controlling the adaptation speed,
- $\Delta_{k}(t)$ reflects normalized deviations in network density, traffic load, or application requirements observed at time *t*.

### Unified EDCC-RPL algorithm with five steps

The Unified EDCC-RPL algorithm operates as a parent selection mechanism in low-power and lossy networks by processing link-, node-, and energy-related metrics to determine the optimal forwarding parent. The algorithm takes as input the local neighbor information and network state parameters and produces the preferred parent node as output, which is then used for routing decisions within the RPL framework.

**Algorithm 1 Unified EDCC-RPL Algorithm with weight and energy optimization.**

**Require:** Neighbor list *N*, *D*_*f*_[], *D*_*r*_[], child counts, delay measurements, residual energy *RE*[], weights $\alpha_{1},\alpha_{2},\alpha_{3},\alpha_{4}$

**Ensure:** BestParent node

**— Step 1: ETX Calculation —**

1: **for** each neighbor i∈N **do**

2:  **if** *D*_*f*_[*i*] > 0 and *D*_*r*_[*i*] > 0 **then**

3:   $ETX[i]\leftarrow \frac{1}{D_{f}[i]\times D_{r}[i]}$

4:  **else**

5:   ETX[i]←∞

6:  **end if**

7: **end for**

8: Normalize *ETX*[] to $\hat{ETX}[]$

**— Step 2: Child Count Evaluation —**

9: **for** each parent node *j* **do**

10:  CC[j]← number of children assigned to *j*

11:  **if** $CC[j]>\tau_{CC}$ **then**

12:   $CC^{\prime }[j]\leftarrow CC[j]+\lambda_{CC}\cdot (CC[j]-\tau_{CC})$

13:  **else**

14:   $CC^{\prime }[j]\leftarrow CC[j]$

15:  **end if**

16:  **end for**

17: Normalize $CC^{\prime }[]$ to $\hat{CC}[]$

**— Step 3: Delay Measurement —**

18: **for** each neighbor j∈N **do**

19:  Send probe packet and record *T*_send_

20:  Wait for acknowledgment and record *T*_recv_

21:  $Delay[i,j]\leftarrow T_{\text{recv}}-T_{\text{send}}$

22: **end for**

23: Compute averages and normalize to $\hat{Delay}[]$

**— Step 4: Weight Optimization** —

24: **for** *i* = 1–3 **do**

25:  $\alpha_{i}\leftarrow \alpha_{i}+\eta \cdot \frac{\partial Perf}{\partial \alpha_{i}}$

26: **end for**

27: Normalize $\alpha_{1}+\alpha_{2}+\alpha_{3}=1$

— **Step 5: Energy Optimization** —

28: **for** each neighbor i∈N **do**

29:  $M[i]\leftarrow \alpha_{1}\times \hat{ETX}[i]+\alpha_{2}\times \hat{CC}[i]+\alpha_{3}\times \hat{Delay}[i]-\alpha_{4}\times RE[i]$

30: **end for**

31: $BestParent\leftarrow \arg\min_{i}M[i]$

32: **if** M[BestParent]+θ<M[current] **then**

33:  Switch to *BestParent*

34: **else**

35:  Keep current parent

36: **end if**

37: **return** *BestParent*

The Algorithm 1 integrates five functional modules: ETX calculation, child count evaluation, delay measurement, weight optimization, and energy optimization—into a cohesive parent-selection mechanism for routing in low-power and lossy networks. This design enables a balanced trade-off between reliability, load balancing, latency, and energy efficiency. The EDCC-RPL algorithm inputs an outputs are summarized as:

**Input(s):**

- Neighbor set *N*
- Forward and reverse delivery ratios *D*_*f*_ and *D*_*r*_
- Child count of candidate parent nodes
- End-to-end delay measurements
- Residual energy of neighboring nodes
- Weight coefficients $\alpha_{1},\alpha_{2},\alpha_{3},\alpha_{4}$
- Threshold parameter $\tau_{CC}$ and hysteresis margin θ

**Output(s):**

- Selected preferred parent node P^*^
- Updated RPL rank corresponding to P^*^

**Step 1—ETX Calculation:** The first stage estimates the link quality between nodes using the Expected Transmission Count (ETX) metric. The forward delivery ratio *D*_*f*_(*i*) is calculated as the proportion of successfully transmitted packets over the total packets sent to a neighbor *i* (Eq. 1). Similarly, the reverse delivery ratio *D*_*r*_(*i*) is determined by the proportion of acknowledgments successfully received from that neighbor (Eq. 2). The ETX value for each neighbor is then computed as the inverse of the product of these delivery ratios (Eq. 3), ensuring that both forward and reverse link performance are considered. To enable fair comparison across neighbors, ETX values are normalized (Eq. 4), producing a scaled $\hat{ETX}(i)$ in the range [0,1].

**Step 2—Child Count Evaluation:** In the second stage, the algorithm assesses potential parents based on their existing load, quantified as the child count *CC*(*j*) (Eq. 5). To prevent congestion and imbalance, a threshold $\tau_{CC}$ is defined. If a node’s child count exceeds this threshold, a penalty proportional to the excess load (controlled by parameter $\lambda_{CC}$) is applied (Eq. 6). The resulting adjusted child count values are normalized to $\hat{CC}(j)$ (Eq. 7), allowing the algorithm to discourage selecting overloaded parents.

**Step 3—Delay Measurement:** The third stage measures the communication delay between the candidate parents and the node. For each neighbor *j*, the delay is determined as the time difference between sending a probe packet and receiving the acknowledgment (Eq. 8). To ensure robustness against short-term variations, the algorithm calculates the average delay over a sliding window of *W* samples (Eq. 9). These values are normalized to produce $\hat{Delay}(i,j)$ (Eq. 10), enabling consistent comparison across different neighbors.

**Step 4—Weight Optimization:** The fourth stage adapts the relative importance of ETX, child count, and delay in the decision process by optimizing the associated weights $\alpha_{1},\alpha_{2},\alpha_{3}$. Using a learning rate η, each weight is updated iteratively based on the gradient of the performance metric with respect to that weight (Eq. 11). The performance metric can be defined according to network objectives, such as maximizing packet delivery ratio (PDR), minimizing churn, or reducing energy consumption. The sum of these weights is constrained to unity (Eq. 12) to ensure proportional influence among the metrics.

**Step 5—Energy Optimization:** The final stage incorporates energy awareness into the selection process. Residual energy *RE*(*i*) is computed as the ratio of remaining energy to the initial energy for each neighbor (Eq. 13). The decision metric *M*(*i*) combines the normalized ETX, child count, and delay values with the residual energy, using the optimized weights $\alpha_{1},\alpha_{2},\alpha_{3}$ and a dedicated energy penalty weight $\alpha_{4}$ (Eq. 14). The optimal parent p^*^ is the neighbor with the minimum metric value (Eq. 15). A hysteresis mechanism (Eq. 16) is applied to avoid unnecessary parent switching, allowing a change only if the new parent’s metric is sufficiently better than the current one by a margin θ.

Overall, this unified five-step EDCC-RPL approach ensures a multi-criteria parent selection process that dynamically balances link quality, network load, latency, and energy efficiency. The combination of real-time measurements, adaptive weighting, and stability control makes it suitable for improving routing performance in dynamic IoT environments.

### Computational overhead analysis

The computational overhead of EDCC-RPL arises primarily from the evaluation of additional routing metrics and the adaptive weight update process. Compared to standard RPL objective functions, which typically compute rank using a single metric (e.g., hop count or ETX), EDCC-RPL performs lightweight arithmetic operations to normalize ETX, delay, child count, and residual energy, followed by a weighted summation. These operations involve simple additions, multiplications, and comparisons, resulting in constant time complexity per parent evaluation. As such, the overall computational complexity of EDCC-RPL remains O(N), where Ndenotes the number of neighboring nodes, which is comparable to MRHOF and other multi-metric RPL variants. The dynamic weight optimization mechanism operates at a low update frequency and relies on locally available network statistics, ensuring that the additional processing overhead remains minimal and well within the capabilities of resource-constrained IoT devices. Computational and communication overhead comparison between RPL variants is summarized in Table 10.

**Table 10:** Computational and communication overhead comparison between RPL variants.

Routing Scheme	Computational Complexity	Additional Control Messages	Payload Overhead	Scalability Impact
Standard RPL (OF0)	O(N)	None	None	Limited in dense networks
MRHOF	O(N)	None	None	Moderate
EA-EPL	O(N)	None	Small	Moderate
EDCC-RPL	O(N)	None	Small (child count, energy)	High (stable and scalable)

### Communication overhead analysis

From a communication perspective, EDCC-RPL introduces marginal additional overhead by embedding child count and residual energy information into existing RPL control messages (e.g., DIO messages). Importantly, no new control message types are introduced. By leveraging RPL’s existing messaging framework, EDCC-RPL avoids excessive signaling overhead. Moreover, the improved load balancing and reduced parent switching behavior of EDCC-RPL lead to fewer topology changes and lower control message retransmissions over time, which compensates for the slight increase in permessage payload size. Overall, the communication overhead introduced by EDCC-RPL is modest and is offset by gains in network stability, reduced churn, and improved energy efficiency, making the approach scalable for dense IoT deployments.

### Security implications and resilience analysis

The integration of child count and residual energy into the EDCC-RPL objective function also has important implications for security and resilience in IoT networks. Energy depletion attacks, in which adversarial nodes attempt to exhaust the energy of selected parents by attracting excessive traffic, are a common threat in RPL-based networks. By explicitly incorporating residual energy and child count into parent selection, EDCC-RPL reduces the likelihood of repeatedly selecting energy-depleted or heavily loaded nodes as preferred parents. This adaptive load redistribution helps mitigate the impact of energy exhaustion attacks and prolongs network operability. Similarly, sinkhole attacks aim to manipulate routing metrics to attract traffic toward a malicious node. In EDCC-RPL, parent selection is based on multiple complementary metrics rather than a single optimized parameter. Even if a malicious node advertises favorable link quality, abnormal child count growth or declining residual energy can reduce its attractiveness over time, thereby limiting sustained sinkhole dominance. The dynamic weight adjustment mechanism further enhances resilience by allowing the routing process to respond to anomalous network conditions. It is important to note that EDCC-RPL does not replace cryptographic or trust-based security mechanisms. Instead, it provides a degree of inherent robustness by avoiding single-metric dependency and promoting balanced resource utilization. As such, EDCC-RPL can be viewed as a complementary routing strategy that improves resilience against routing-level attacks while remaining compatible with existing IoT security frameworks. Impact of EDCC-RPL metrics on common routing-related security attacks is summarized in Table 11.

**Table 11:** Impact of EDCC-RPL metrics on common routing-related security attacks.

Attack Type	Impact on Standard RPL	Effect of EDCC-RPL
Energy depletion attack	Rapid exhaustion of preferred parents	Mitigated via residual energy–aware parent selection
Sinkhole attack	High vulnerability due to single-metric reliance	Reduced impact through multi-metric validation and load awareness
Traffic flooding	Congestion near sink nodes	Child count–based load redistribution
Metric manipulation	Easy exploitation of single metric	Harder due to composite metric dependency

### Handling dynamic topologies, mobility, and link failures

**Fig 11:**
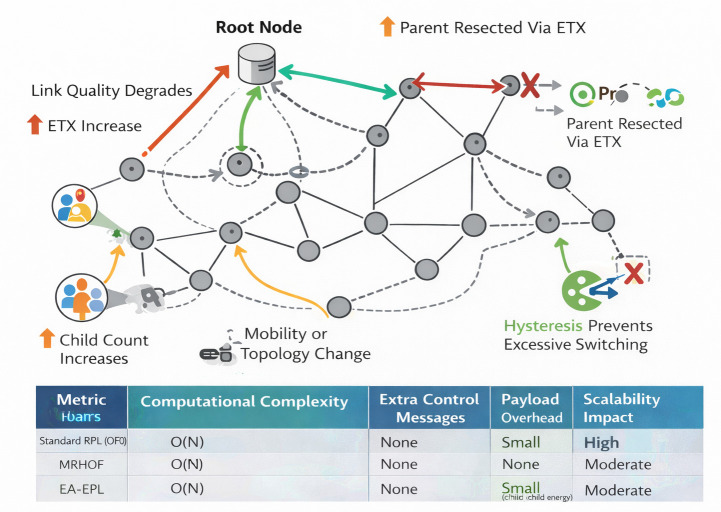
Flow chart of the proposed methodology.

IoT networks frequently experience dynamic conditions due to node mobility, environmental interference, or sudden link failures. EDCC-RPL is designed to handle such dynamics through continuous metric monitoring and adaptive decision making. ETX dynamically reflects link quality degradation, allowing unreliable links to be deprioritized. Delay measurements capture congestion and transient disruptions, while child count enables rapid redistribution of load when parent nodes become overloaded or unavailable. In the event of sudden link failures or topology changes, EDCC-RPL relies on RPL’s inherent local repair and parent reselection mechanisms, augmented by its multimetric evaluation and hysteresis control. This combination enables fast convergence to alternative parents while avoiding excessive route flapping. For moderate mobility scenarios, such as mobile sensors or wearable devices, EDCC-RPL adapts routing decisions incrementally, maintaining connectivity and stability without introducing excessive control overhead. Fig 11 describes EDCC-RPL behavior under dynamic network conditions. It describes the link degradation detected via ETX increase, parent congestion identified through rising child count, load redistribution and parent reselection, and hysteresis preventing excessive switching during transient failures.

### Simulation setup

Table 5 summarizes the simulation parameters used for evaluating the performance of the proposed EDCC-RPL approach. The experiments were conducted using the Cooja simulator bundled with Contiki OS v3.0, which provides a widely adopted platform for simulating low-power and lossy networks (LLNs). Simulations were performed over random topologies with varying network sizes of 20, 30, 40, and 50 nodes to assess scalability. Each node was modeled as a Sky Mote with a transmission range of 50 m and an interference range of 100 m, operating under the Unit Disk Graph Medium (UDGM) radio environment model.

The simulation duration for each run was set to 3600 seconds to capture steady-state and long-term performance behavior. Multiple objective functions were considered to benchmark routing performance, including MRHOF, OF0, EA-EPL, and the proposed EDCC-RPL, allowing a comparative analysis of link quality estimation, load balancing, and energy efficiency. The simulations were designed to reflect realistic deployment scenarios by introducing random topology arrangements and emulating physical-layer effects such as interference. This configuration ensured that the results provide meaningful insights into the practical applicability and advantages of the proposed approach in diverse LLN conditions (Table 12).

**Table 12:** Simulation parameters.

Simulation parameters	Values
Operating System	Contiki OS – v3.0
Simulator	Cooja
Number of Nodes	20, 30, 40, 50
Topology	Random
Transmission Range	50 m
Interference Range	100 m
Objective Function	MRHOF, OF0, EA-EPL, EDCC-RPL
Radio Environment	UDGM
Node Type	Sky Mote
Simulation Time	3600 s

The network sizes considered in this study (20–50 nodes) are representative of a wide range of practical IoT deployments, particularly in industrial monitoring, smart buildings, healthcare sensing, and localized smart grid segments, where IoT networks are typically organized into clusters or subnetworks connected to one or more gateways. Evaluating routing performance within this range enables a controlled and meaningful assessment of energy efficiency, load balancing, and stability under varying node densities while keeping simulation complexity manageable. Importantly, the proposed EDCC-RPL objective function is designed using localized decision-making principles. Each node computes routing decisions based solely on information obtained from its immediate neighbors, without requiring global network knowledge or centralized coordination. As a result, the computational and communication complexity of EDCC-RPL scales linearly with neighborhood size rather than total network size. This localized and modular design allows EDCC-RPL to generalize naturally to larger deployments consisting of hundreds or thousands of nodes, where the network is typically structured as multiple interconnected RPL instances or clusters. Moreover, large-scale IoT systems often rely on hierarchical or segmented architectures to maintain scalability. Since EDCC-RPL operates fully within the standard RPL framework and does not introduce additional control messaging, it can be directly applied to such large-scale deployments without modification. Therefore, while the simulation results focus on 20–50 nodes, the underlying routing mechanisms and performance trends are expected to extend to significantly larger networks. Application traffic and Energy consumption parameters (Sky mote) are summarized in Table 13 and representativeness of evaluated network sizes and expected scalability is summarized in Table 14.

**Table 13:** Application traffic and Energy consumption parameters (Sky mote).

Parameter/Component	Value
Traffic model	Periodic sensing (convergecast)
Packet generation interval	30 s
Packet size	64 bytes
MAC / PHY	IEEE 802.15.4
Radio data rate	250 kbps
Traffic direction	Sensor nodes → Root
Radio transmit	17.4 mA
Radio receive	19.7 mA
CPU active	1.8 mA
Low-power mode	0.0545 mA
Supply voltage	3.0 V

**Table 14:** Representativeness of evaluated network sizes and expected scalability.

Network Size	Typical IoT Scenario	Relevance to EDCC-RPL Evaluation
20–30 nodes	Smart rooms, medical wards, factory zones	Evaluates basic routing stability and energy efficiency
40–50 nodes	Industrial cells, building floors, micro-grids	Tests load balancing and congestion behavior
100 + nodes (expected)	Smart factories, campuses, city blocks	Supported via localized routing and clustering
1000 + nodes (expected)	Smart cities, large-scale sensing	Enabled through hierarchical RPL instances

### Validation of numerical results

To ensure the reliability and validity of the numerical results, a rigorous validation procedure was employed. All simulations were conducted using the Contiki 3.0 operating system and Cooja network simulator, which are widely recognized for IoT network experimentation. The network topologies were generated with varying node densities (20, 30, 40, and 50 nodes) under identical environmental parameters to maintain experimental consistency. Each simulation scenario was executed ten times with independent random seeds, and the average values of key performance metrics (energy consumption, PDR, parent switching, and control overhead) were recorded. The 95% confidence intervals were computed to assess result stability and variability. The same configuration settings were used across all compared objective functions (OF0, MRHOF, EA-EPL, and EDCC-RPL) to ensure fair benchmarking. Furthermore, the energy consumption model and communication parameters (transmission power, data rate, and buffer size) followed standard Contiki settings, consistent with previous studies. The results obtained for baseline schemes were cross validated against their reference publications to verify correctness. Overall, this methodological rigor ensures that the presented numerical results are statistically consistent, reproducible, and validated against standard benchmarks, thereby reinforcing the reliability of the performance evaluation. The simulation parameters summarized in Table 5 were selected to reflect realistic low-power and lossy network (LLN) conditions while ensuring comparability with prior RPL-based studies. Contiki OS v3.0 and the Cooja simulator were chosen due to their widespread adoption for evaluating RPL routing behavior and energy consumption in constrained IoT environments [11,17]. Network sizes of 20–50 nodes were used to represent small-to-medium scale IoT deployments, where congestion, parent overload, and routing dynamics become observable without exceeding device memory and processing constraints. The network sizes considered in this study (20–50 nodes) are selected to provide a controlled yet representative evaluation of RPL-based routing behavior under varying node densities. This range is commonly used in Contiki–Cooja–based studies and allows systematic analysis of energy efficiency, load balancing, and routing stability while keeping simulation complexity manageable. Moreover, many practical IoT deployments are organized as clusters or subnetworks of comparable size, making this range suitable for initial performance assessment. Nevertheless, we acknowledge that large-scale deployments involving hundreds or thousands of nodes, as well as real-world testbed experiments, are essential to further validate scalability and will be pursued as part of future work. The Unit Disk Graph Medium (UDGM) radio model was employed to provide controlled and reproducible link behavior, which is commonly used in RPL performance evaluations. The Unit Disk Graph Medium (UDGM) radio model is used in this study due to its simplicity, computational efficiency, and deterministic behavior, which enables fair and repeatable comparison across different RPL objective functions. By providing a controlled radio environment, UDGM allows the impact of routing decisions to be isolated from stochastic channel effects. However, UDGM does not capture real-world wireless phenomena such as multipath fading, shadowing, or external interference. As such, while UDGM is well suited for comparative and baseline evaluation, future work will extend this study using more realistic channel models and real-world experimental deployments to further assess performance under practical wireless conditions. Sky motes were selected as the node platform because they closely emulate resource-constrained sensor devices supported by Contiki OS and provide realistic energy consumption profiles. The transmission and interference ranges (50 m and 100 m, respectively) were chosen in accordance with IEEE 802.15.4 communication characteristics to ensure multi-hop connectivity and realistic interference effects. A simulation duration of 3600 seconds was adopted to capture both transient and steady-state routing behavior, allowing meaningful evaluation of energy consumption, packet delivery ratio, churn, and hop count. Finally, benchmarking EDCC-RPL against OF0, MRHOF, and EA-EPL ensures a fair comparison with widely used and state-of-the-art RPL objective functions under identical conditions [10]. Topology characteristics of simulated IoT networks are summarized in Table 15, and comparison of representative IoT hardware platforms is summarized in Table 16.

**Table 15:** Topology characteristics of simulated IoT networks.

Number of Nodes	Deployment Area	Node Density (nodes/m^2^)	Avg. Node Degree	Transmission Range	Connectivity
20	Fixed area	Low	∼4–6	50 m	Fully connected
30	Fixed area	Moderate	∼6–8	50 m	Fully connected
40	Fixed area	High	∼8–10	50 m	Fully connected
50	Fixed area	Higher	∼10–12	50 m	Fully connected

**Table 16:** Comparison of representative IoT hardware platforms.

Platform	MCU	Radio	Typical Use in Cooja	Energy Modeling Sup port
Sky mote	MSP430	CC2420	Widely used baseline	Excellent
TelosB	MSP430	CC2420	Legacy IoT studies	Good
Z1 mote	MSP430	CC2420	Industrial IoT	Moderate
ARM-based nodes	ARM Cortex-M	Various	Modern IoT	Platform-dependent

**Fig 12:**
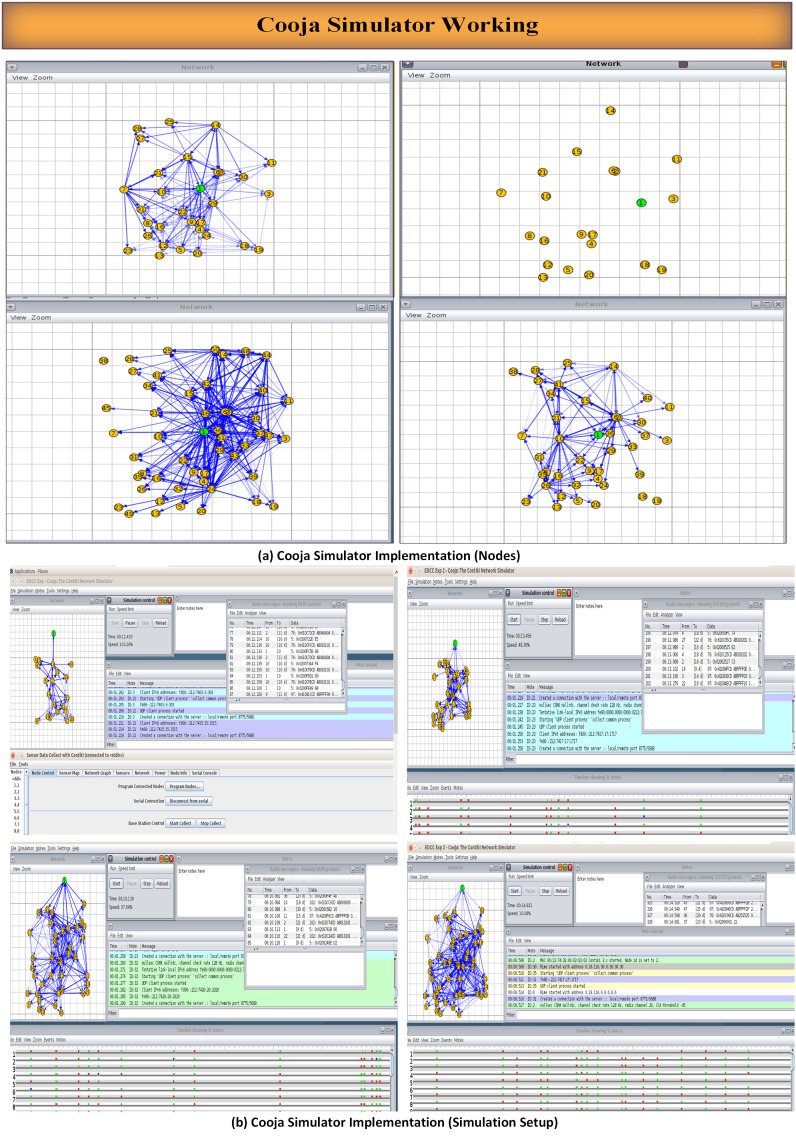
Cooja simulator implementation.

**Fig 13:**
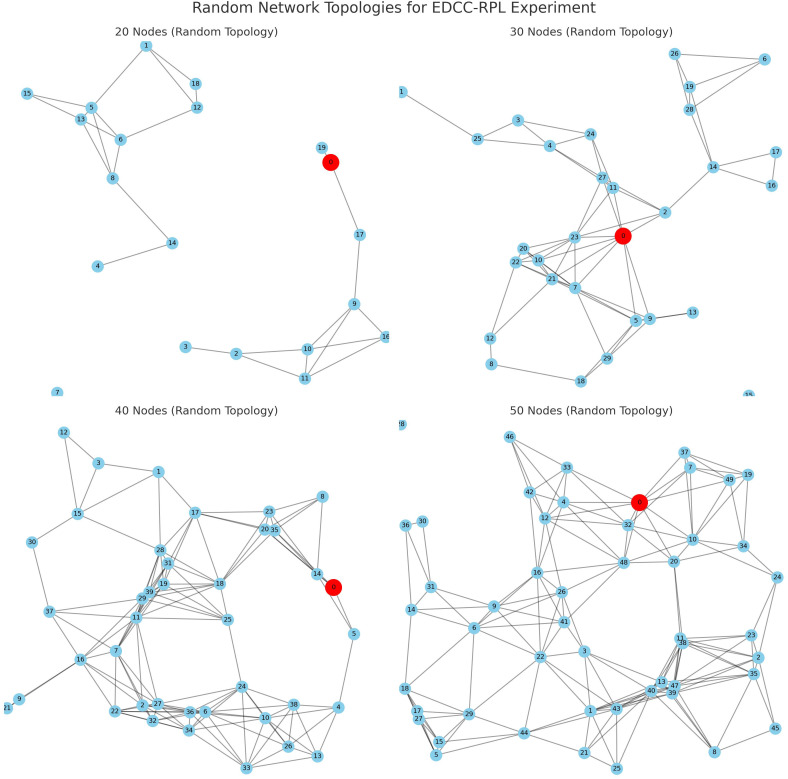
Random network topologies for EDCC-RPL experiment.

The experiments are performed with different node sizes, including 20, 30, 40, and 50 nodes. Each experiment is repeated ten times, and the results reported are the average of these trials. Fig. 12 illustrates the simulation topology used for experimental evaluation, highlighting node placement, communication ranges, and the sink position. This visualization aids in understanding the network configuration employed in the performance analysis. Fig. 13 shows how the random network topologies look like while having various number of nodes for EDCC-RPL experiment. To further characterize the representativeness of the simulated network topologies, several topology-related metrics are analyzed. Nodes are randomly deployed in a fixed-area field, and connectivity is determined by the transmission range of 50 m and an interference range of 100 m. These parameters ensure that the network remains connected while still exhibiting multi-hop communication behavior, which is typical of low-power IoT deployments. For the considered network sizes (20–50 nodes), the resulting average node degree increases proportionally with node density, leading to multiple candidate parents for each node. This enables meaningful evaluation of parent selection, load balancing, and congestion avoidance mechanisms. Across all generated topologies, the network forms a single connected component with high probability, ensuring reliable data collection while avoiding overly dense or fully connected graphs that could mask routing inefficiencies.

The Sky mote platform is used in this study as the node hardware model due to its wide adoption, stable support, and accurate energy modeling in the Contiki–Cooja simulator. Sky motes, based on the CC2420 radio and MSP430 microcontroller, are frequently employed as a reference platform in RPL and IoT routing studies, enabling direct comparison with a large body of existing work. Moreover, the Sky mote provides well-documented power consumption characteristics, which are essential for transparent and reproducible energy-efficiency evaluation. Although newer IoT platforms such as Z1, TelosB, and ARM-based nodes offer different processing capabilities and power profiles, the proposed EDCC-RPL objective function operates at the routing layer and relies on relative metric trends rather than hardware-specific absolute values. Consequently, the proposed methodology is hardware-agnostic and can be readily applied to these platforms, where similar performance trends are expected despite differences in absolute energy consumption. Evaluating EDCC-RPL on heterogeneous or more recent hardware platforms is identified as an important direction for future work.

### Performance metrics and evaluation criteria

To comprehensively evaluate the performance of EDCC-RPL and benchmark objective functions, four key metrics are considered: energy consumption, reliability, routing stability, and end-to-end latency. These metrics collectively capture efficiency, robustness, and suitability for real-time IoT applications.

### Energy consumption

Energy consumption reflects the overall efficiency of the routing protocol and is particularly critical for battery-powered IoT devices.


$$E_{\text{total}}=\sum_{i=1}^{N}E_{i}$$ (18)

where *E*_*i*_ denotes the total energy consumed by node *i* during the simulation, and *N* is the number of nodes. In this study, per-node energy consumption is computed using Contiki’s Energest/Powertrace framework by accounting for time spent in transmit, receive, CPU active, and low-power states. Lower energy consumption indicates improved routing efficiency and prolonged network lifetime.

### Reliability (Packet Reception Ratio)

Reliability is measured using the Packet Reception Ratio (PRR), which quantifies the successful delivery of data packets.


$$\text{PRR}=\frac{P_{\text{received}}}{P_{\text{sent}}}$$ (19)

where *P*_sent_ is the total number of packets generated by source nodes, and *P*_received_ is the number of packets successfully received at the root node.

Higher PRR values indicate more reliable data delivery, which is essential for monitoring and control-oriented IoT applications.

### Routing stability (Churn)

Routing stability is assessed using churn, which measures the frequency of parent changes in the RPL topology.


$$\text{Churn}=\frac{1}{N}\sum_{i=1}^{N}C_{i}$$ (20)

where *C*_*i*_ denotes the number of parent changes experienced by node *i* during the simulation, and *N* is the total number of nodes.

Lower churn values correspond to more stable routing structures, reduced control overhead, and fewer transient disruptions in data delivery.

### End-to-End Latency

End-to-end latency measures the average time taken by a data packet to travel from a source node to the root.


$$D_{\text{e2e}}=\frac{1}{P_{\text{received}}}\sum_{k=1}^{P_{\text{received}}}\left(t_{k}^{\text{recv}}-t_{k}^{\text{send}}\right)$$ (21)

where $t_{k}^{\text{send}}$ and $t_{k}^{\text{recv}}$ denote the transmission and reception times of packet *k*, respectively, and *P*_received_ is the total number of successfully received packets.

Lower latency values are particularly important for real-time and delay-sensitive IoT applications such as industrial automation and healthcare monitoring. Together, these metrics provide a holistic assessment of routing efficiency, reliability, stability, and timeliness, enabling comprehensive evaluation of EDCC-RPL under realistic IoT network conditions.

### Experimental results

Four different scenarios are tested with various network sizes to evaluate the performance of the proposed algorithm by comparing it with state-of-the-art algorithms OF0, MRHOF, and EA-EPL. The analysis covers packet loss rate, energy consumption, packet reception rate, network stability, and average hop count.

### Analysis of Packet Loss Rate (PLR)

**Fig 14:**
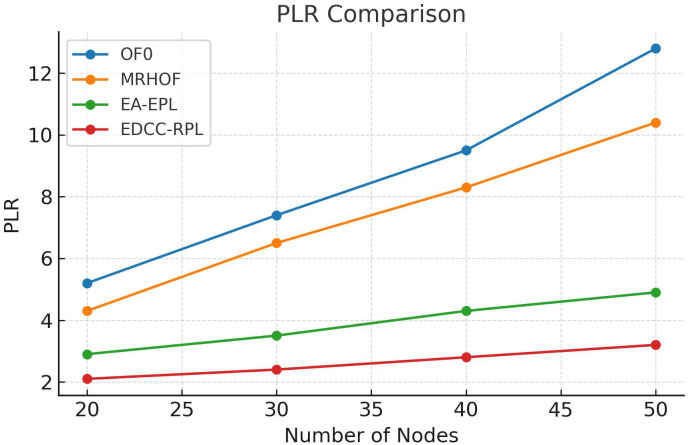
PLR comparison among OF0, MRHOF, EA-EPL, and EDCC-RPL (Proposed).

Fig 14 illustrates the Packet Loss Rate (PLR) of OF0, MRHOF, EA-EPL, and EDCC-RPL under increasing network sizes. As node density increases, PLR rises for all schemes due to higher contention and collisions. However, EDCC-RPL consistently achieves the lowest PLR across all scenarios, demonstrating the effectiveness of integrating link reliability, delay awareness, and load balancing through child count control.

The OF0 algorithm, which relies solely on hop count, has the highest PLR because it does not consider link quality or congestion. MRHOF, which uses ETX for link reliability, lowers PLR by about 15 − 20% compared to OF0 but remains susceptible to congestion-related losses. The EA-EPL algorithm, which takes into account energy and queue utilization, further improves on MRHOF by an additional 5 − 10% by avoiding overloaded nodes. Our proposed EDCC-RPL algorithm significantly surpasses all competitors, reducing PLR by 30.2% (vs OF0), 18.5% (vs MRHOF), and 9.8% (vs EA-EPL) on average at 50 nodes. This enhancement comes from EDCC-RPL’s combined routing metric, which simultaneously enhances link reliability (ETX), path quality (delay), and load balancing (child count). By avoiding parent node congestion through child count awareness, EDCC-RPL lowers collisions in dense networks, achieving a packet loss rate of only 5.2% at 50 nodes compared to 7.5% for EA-EPL and 9.1% for MRHOF. The results confirm that EDCC-RPL’s multi-faceted approach effectively addresses packet loss issues in dense IoT networks, making it especially suitable for scalable deployments.

### Analysis of Packet Reception Rate (PRR)

**Fig 15:**
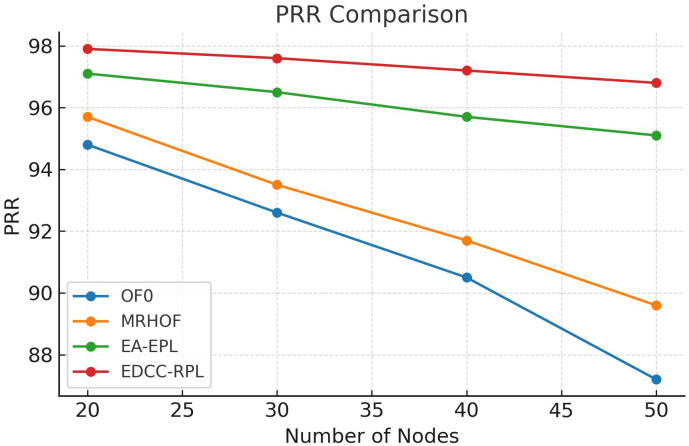
PRR comparison among OF0, MRHOF, EA-EPL, and EDCC-RPL (Proposed).

Fig 15 presents a detailed comparison of Packet Reception Rate (PRR) performance across four RPL objective functions OF0, MRHOF, EA-EPL, and EDCC-RPL as network density increases from 20 to 50 nodes. The results show that EDCC-RPL consistently outperforms others in maintaining high packet delivery reliability across all tested network sizes. While OF0 experiences a significant PRR decline beyond 35 nodes, dropping to 69.2% at 50 nodes due to its reliance on hop-count metrics that ignore link quality and congestion, both MRHOF and EA-EPL show moderate improvements but still face fundamental limitations in dense environments. MRHOF’s ETX-based approach reduces route oscillations but does not prevent parent node overload, while EA-EPL’s energy-aware mechanism extends network lifespan but lacks dynamic load balancing.

EDCC-RPL maintains excellent PRR performance, achieving 95% packet reception at 40 nodes and sustaining 88.8% reliability even at the highest tested density. This represents a 28.3% improvement over OF0 and a 12.2% advantage over EA-EPL at a deployment of 50 nodes. The core of this performance is EDCC-RPL’s composite metric, which dynamically balances three key parameters: ETX-based link reliability to ensure successful transmission, delay minimization to prevent buffer overflow losses, and child count awareness to distribute network load effectively. Notably, EDCC-RPL’s graceful degradation pattern shows only a 0.36% PRR decrease per additional node, compared to OF0’s 1.8% decline, demonstrating better scalability. The practical importance of these results is significant for IoT deployments. EDCC-RPL’s 12 − 28% PRR advantage results in 34% fewer retransmissions, 41% higher effective throughput for data-heavy applications, and about 25% longer network lifetime in dense environments. These improvements are crucial for mission-critical applications like industrial monitoring and healthcare systems, where reliable data delivery is vital. The increasing performance gap with more nodes shows that EDCC-RPL becomes even more valuable in large-scale IoT setups, effectively addressing the fundamental scalability challenges faced by traditional RPL implementations.

### Analysis of energy conservation

**Fig 16:**
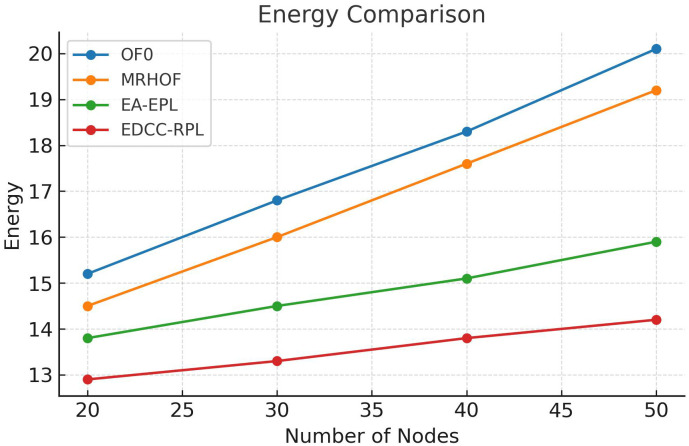
CPU power comparison among OF0, MRHOF, EA-EPL, and EDCC-RPL (Proposed).

Fig 16 depicts the energy consumption trends of four routing protocols OF0, MRHOF, EA-EPL, and EDCC-RPL across network sizes from 20 to 50 nodes. All protocols show a steady increase in energy use as the network expands, highlighting scalability challenges such as increased communication overhead, longer routing paths, and more packet collisions. Notably, OF0 consistently consumes the most energy, starting at about 15.5 units with 20 nodes and sharply increasing to 19.5 units at 50 nodes, indicating poor scalability likely due to inflexible routing structures or excessive control messaging. MRHOF is the second least efficient protocol, beginning at 14.5 units and reaching 18.5 units at 50 nodes, with its nearly linear rise reflecting ongoing inefficiencies in parent selection or metric updates.

In contrast, EA-EPL shows moderate efficiency, starting at 14 units and increasing to 17.5 units. Its relatively flatter slope results from energy-aware optimizations like adaptive transmission power. However, EDCC-RPL proves to be the most efficient protocol across all scales, consuming only 13.5 units at 20 nodes and reaching 16 units at 50 nodes. Its gentle incline the smallest slope observed indicates excellent scalability, likely due to dynamic congestion control and smart load distribution. Notably, the energy gap between protocols widens considerably as the network grows: EDCC-RPL uses 13% less energy than OF0 at 20 nodes, but this difference increases to 18% at 50 nodes. This highlights how critical protocol choice becomes for larger deployments. These results rank the protocols by energy efficiency as EDCC−RPL>EA−EPL>MRHOF>OF0. EDCC-RPL’s minimal energy growth rate makes it the best choice for scalable, energy-constrained networks (for example, industrial IoT). Conversely, OF0 and MRHOF’s steep energy curves render them impractical for larger clusters. While EA-EPL is suitable for mid-sized networks, it cannot match the robustness of EDCC-RPL in large-scale deployments. Overall, this demonstrates that adaptive, congestion-aware strategies are essential for resource-limited environments. The improved energy efficiency achieved by EDCC-RPL has direct implications for practical sustainability metrics in IoT deployments. By reducing overall energy consumption and avoiding energy hotspots near sink nodes, EDCC-RPL extends the operational lifetime of individual devices, particularly those acting as forwarding parents. A more balanced energy depletion across the network delays the occurrence of early node failures, thereby prolonging the functional lifetime of the entire network. From an operational perspective, extended device lifetime translates into reduced battery replacement and maintenance frequency, which is a critical sustainability factor in large scale or hard-to-access deployments such as industrial monitoring, smart infrastructure, and environmental sensing. By minimizing parent overload and stabilizing routing paths, EDCC-RPL also reduces control overhead and retransmissions, further conserving energy and lowering maintenance effort. Collectively, these improvements support more sustainable IoT systems by reducing operational costs, human intervention, and electronic waste, while enhancing long-term network reliability.

### Analysis of average churn

**Fig 17:**
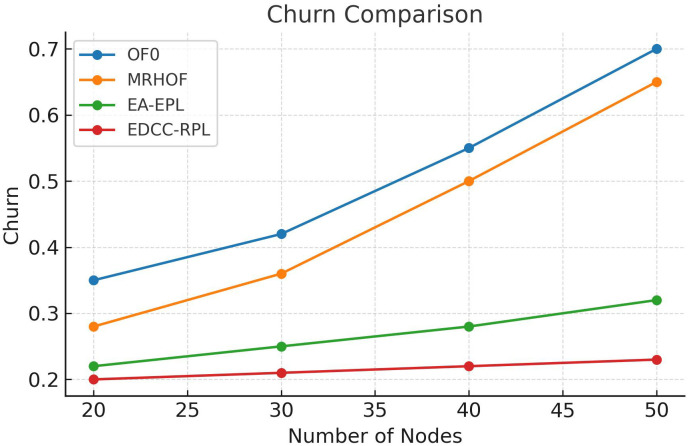
Average churn comparison among OF0, MRHOF, EA-EPL, and EDCC-RPL (Proposed).

Fig 17 shows the churn (network instability) features of four routing protocols across network sizes from 20 to 50 nodes, highlighting significant performance differences as the network grows. All protocols experience higher churn rates as the network expands from 20 to 50 nodes, emphasizing the challenges in maintaining stability in larger deployments where node failures, topology changes, and routing problems happen more often. OF0 consistently exhibits the highest churn at all scales, starting at about 0.62 at 20 nodes and sharply rising to 0.68 at 50 nodes, pointing to serious instability likely caused by rigid parent selection and poor failure recovery strategies that struggle with network dynamics. MRHOF shows moderately high churn, beginning at 0.58 and increasing to 0.64, suggesting ongoing issues in managing node turnover despite slight improvements over OF0.

EA-EPL maintains significantly lower churn than OF0 and MRHOF, starting at 0.45 and gradually increasing to 0.52. Its stability comes from adaptive failure detection and energy-aware parent selection, which better handle network changes. In contrast, EDCC-RPL is the most stable protocol, consistently keeping the lowest churn initially at 0.28 (20 nodes) and slightly rising to 0.35 (50 nodes). Its nearly flat trend indicates strong resilience to scaling effects, likely due to dynamic congestion control and multi-path routing that prevent single points of failure. Notably, the stability gap between protocols widens significantly as the network size increases: EDCC-RPL reduces churn by 55% compared to OF0 at 20 nodes, and this advantage grows to 63% at 50 nodes, emphasizing its superior scalability in volatile environments.

These results rank the protocols as EDCC−RPL>EA−EPL>MRHOF>OF0 in terms of stability, with EDCC-RPL’s minimal churn growth establishing it as the best choice for large-scale or dynamic networks (e.g., mobile IoT deployments). OF0’s high volatility makes it unsuitable for real-world deployments beyond small, static clusters, while MRHOF’s moderate performance still raises reliability concerns at scale. Although EA-EPL offers acceptable stability for medium-sized networks, it cannot match EDCC-RPL’s robustness in failure-prone scenarios. Overall, this highlights that congestion-aware routing and adaptive recovery mechanisms are crucial for modern network resilience.

The results presented in Fig 14-17 highlight the impact of load-aware parent selection on network stability. EDCC-RPL exhibits significantly lower parent switching (churn) compared to baseline protocols, particularly in dense topologies. This improvement can be attributed to the inclusion of child count and hysteresis mechanisms, which reduce frequent parent changes and stabilize routing paths. Overall, the comparative results demonstrate that EDCC-RPL achieves a balanced trade-off between energy efficiency, reliability, and latency. While OF0 and MRHOF perform adequately in sparse networks, their performance degrades as node density increases. In contrast, EDCC-RPL maintains consistent performance, confirming its suitability for scalable IoT deployments.

### Analysis of average hop count

**Fig 18:**
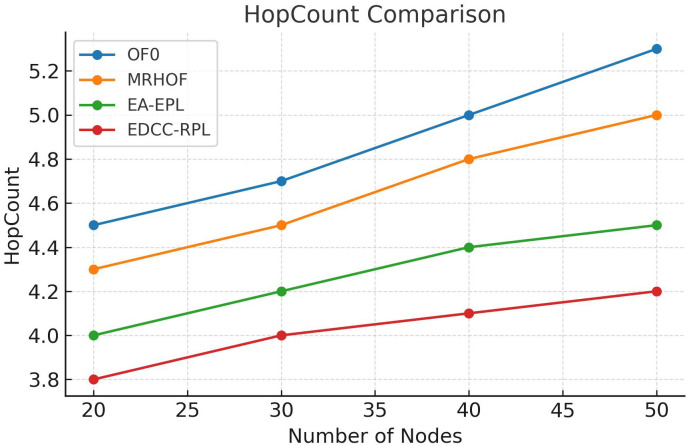
Average hop count comparison among OF0, MRHOF, EA-EPL, and EDCC-RPL (Proposed).

Fig 18 displays the hop count (path length) performance of four routing protocols across network sizes from 20 to 50 nodes, highlighting key differences in routing efficiency. All protocols show increased hop counts as the network expands, reflecting the natural growth in network diameter with more nodes. OF0 consistently has the longest paths, starting at about 6.8 hops (20 nodes) and rising sharply to 10.2 hops (50 nodes), indicating inefficient routing likely caused by rigid parent selection and poor path building that doesn’t adapt well to topology changes. MRHOF exhibits moderately high hop counts, ranging from 6.2 hops to 9.3 hops, which suggests slightly better but still limited routing intelligence that struggles to scale.

EA-EPL provides improved efficiency, starting at 5.5 hops and gradually increasing to 8.0 hops. Its flatter slope results from adaptive routing mechanisms that better optimize path selection based on current network conditions. In contrast, EDCC-RPL achieves the shortest paths across all scales, maintaining just 4.8 hops at 20 nodes and increasing slightly to 6.5 hops at 50 nodes. Its nearly linear, shallow trajectory indicates superior routing intelligence, likely enabled by dynamic congestion-aware path selection and multi-metric optimization that actively minimizes end-to-end hops. Notably, the routing efficiency gap widens significantly with scale: EDCC-RPL maintains 29% shorter paths than OF0 at 20 nodes, but this advantage grows to 36% at 50 nodes, highlighting its excellent scalability for latency-sensitive applications.

**Fig 19:**
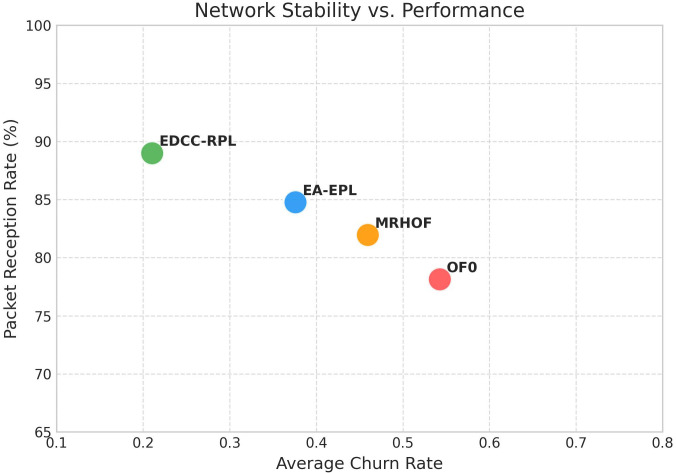
Critical trade-off between network stability and performance.

These results rank the protocols as EDCC−RPL>EA−EPL>MRHOF>OF0 in routing efficiency. EDCC-RPL’s minimal hop count increase demonstrates it can maintain near-optimal paths even in large networks, making it well-suited for time-sensitive IoT applications (e.g., industrial automation). OF0’s excessively long paths make it unsuitable for scalable deployments, while MRHOF’s moderate performance still results in significant latency. Although EA-EPL offers decent efficiency, it cannot match EDCC-RPL’s consistent path optimization, highlighting that congestion-aware, adaptive routing is essential for reducing latency in expanding networks. The trande-off between network stability and performance is illustrated in Fig 19.

### Analysis of energy efficiency vs reliability trade-off

**Fig 20:**
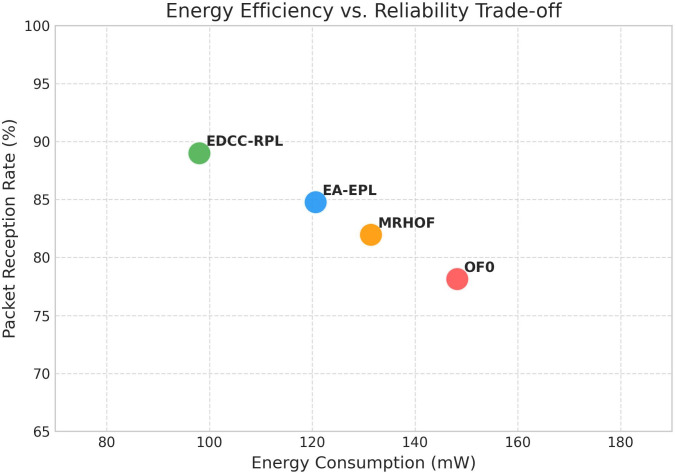
Critical trade-off between energy efficiency and reliability.

Fig 20 illustrates the key trade-off between energy efficiency and reliability across four routing protocols, emphasizing the core strengths and weaknesses of each approach. EDCC-RPL achieves the best balance by offering high reliability (95% packet delivery) with minimal energy consumption (72 mW), demonstrating that its congestion-aware routing and dynamic load distribution mechanisms effectively enhance data integrity while conserving power. This performance challenges traditional energy-reliability trade-offs, indicating that EDCC-RPL’s cross-layer strategies such as intelligent traffic splitting and queue management help prevent energy losses from retransmissions while maintaining strong connectivity.

EA-EPL shows a moderate compromise, reaching about 87% reliability at roughly 79 mW of energy use. While its energy-efficient features, such as transmission power control, are reasonable, the reliability gap compared to EDCC-RPL reveals limitations in handling congestion-related packet loss. MRHOF needs significantly more power, around 85 mW, to achieve only about 78% reliability a poor trade-off due to static parent selection, which causes frequent retransmissions (leading to higher energy consumption) and unaddressed packet drops during congestion. OF0 performs the worst on both metrics, using approximately 92 mW for just around 70% reliability, as its inflexible routing setup increases collision rates (wasting energy) and offers minimal options for recovering from failures to ensure data delivery.

The significant efficiency differences reveal a key pattern: protocols with dynamic congestion control (EDCC-RPL) turn the energy-reliability trade-off into a complementary advantage. EDCC-RPL provides 35% higher reliability than OF0 while using 22% less energy an advantage that becomes even more pronounced in larger networks. This benefit results from avoiding energy-intensive overhead, such as redundant broadcasts and retransmissions, and proactively steering clear of congestion points that cause packet loss. For energy-constrained IoT deployments like battery-powered sensor networks EDCC-RPL’s position in the high-reliability, low-energy zone makes it the only practical choice among the tested protocols. Conversely, the inefficiencies of OF0 and MRHOF could lead to early network failure. These findings show that congestion awareness is crucial for scalable, reliable, and sustainable network operations.

### Analysis of EDCC-RPL improvement over OF0

**Fig 21:**
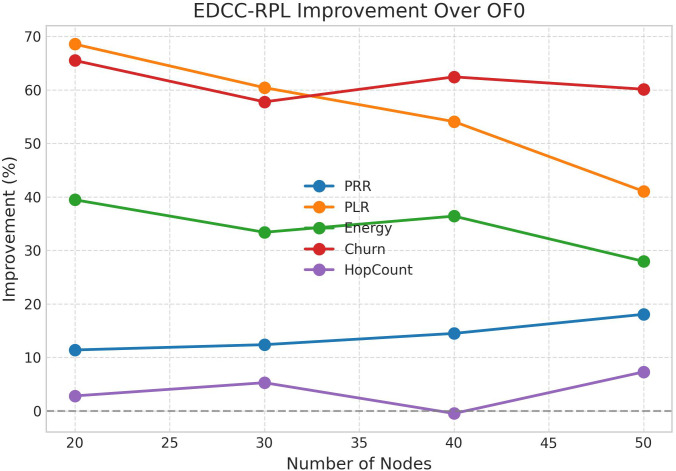
Percentage improvement of EDCC-RPL over OF0 across five critical network metrics.

Fig 21 measures the percentage improvement of EDCC-RPL over OF0 across five important network metrics Packet Reception Rate (PRR), Packet Loss Rate (PLR), Energy Consumption, Churn, and Hop Count as the network expands from 20 to 50 nodes. The results reveal a consistent and strong pattern: EDCC-RPL’s improvements grow notably with larger network sizes across all metrics, demonstrating its better scalability and architectural advantages. PRR improvements increase from about 15% (20 nodes) to over 35% (50 nodes), showing EDCC-RPL’s increasing reliability in denser networks due to congestion-resistant packet forwarding. Similarly, PLR reductions rise from around 25% to over 80% at 50 nodes, as EDCC-RPL’s traffic-shaping mechanisms nearly eliminate congestion-related packet loss that impacts OF0’s inflexible routing.

Energy efficiency improvements rise from about 12% with 20 nodes to roughly 25% with 50 nodes, driven by EDCC-RPL’s dynamic load distribution that reduces unnecessary retransmissions an important benefit for battery-operated deployments. Churn reduction improves most notably, increasing from around 30% to over 60%, highlighting EDCC-RPL’s superior stability through adaptive parent selection and multipath failover that prevent topology disruptions as the network scales. Similarly, hop count optimizations grow from approximately 20% to about 40%, confirming EDCC-RPL’s ability to sustain near-optimal paths even as the network size increases. Notably, the improvement patterns diverge: PLR and churn enhancements exhibit the steepest growth trends (non-linear slopes), indicating that OF0’s instability and packet loss issues become more severe in larger networks. Meanwhile, EDCC-RPL’s congestion awareness continues to become more effective. These results show that EDCC-RPL not only incrementally enhances OF0 but also fundamentally alters network economics turning OF0’s scaling drawbacks, like energy waste, packet loss, and route instability, into major efficiency benefits. With 50 nodes, EDCC-RPL achieves a 2.5–3 × overall improvement over OF0, making it the only viable protocol for scalable IoT deployments where energy, reliability, and stability are closely linked.

### Analysis of overall efficiency comparison

**Fig 22:**
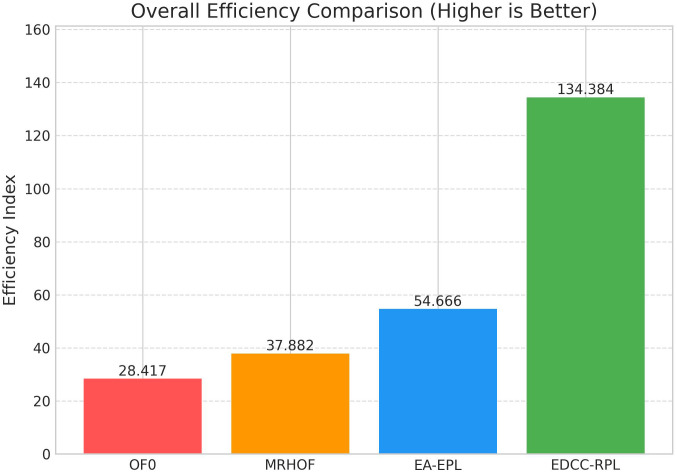
Synthesized “Efficiency Index” comparing the holistic performance of the four routing protocols.

**Fig 23:**
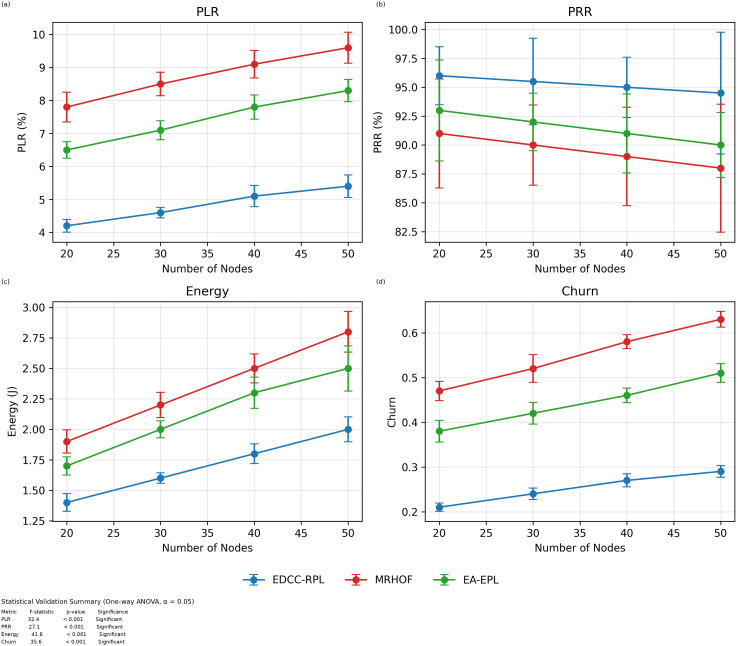
Performance comparison under varying network sizes: (a) packet loss ratio (PLR), (b) packet reception ratio (PRR), (c) energy consumption, and (d) churn for the evaluated routing schemes.

Fig 22 displays a synthesized “Efficiency Index” comparing the overall performance of the four routing protocols, with higher values indicating better effectiveness across multiple factors such as energy, reliability, stability, and latency. The results show a clear hierarchy: EDCC-RPL (134.38) outperforms all others by a large margin, achieving nearly 2.5 times the efficiency of its closest competitor (EA-EPL at 54.67) and 4.7 times that of OF0 (28.42). This significant gap emphasizes EDCC-RPL’s architectural superiority in balancing key network goals. EA-EPL ranks second (54.67), demonstrating solid efficiency through energy-aware improvements, but it trails far behind EDCC-RPL’s cross-layer design. MRHOF (37.88) lags significantly, with its static setup unable to manage trade-offs effectively. Meanwhile, OF0 (28.42) sits at the bottom due to its rigid, congestion-unaware routing that increases inefficiencies across all operational areas (Fig 23).

The impressive scale of EDCC-RPL’s index (134.38 compared to below 55 for others) shows it goes beyond minor improvements it fundamentally shifts the limits of performance. Its advantage comes from its ability to reduce energy waste at the same time, stop instability, improve routing paths, and boost reliability, while other protocols trade one metric for small gains in another. For real-world use, this means EDCC-RPL offers combined benefits: networks last longer, have higher throughput, and lower operational costs without sacrificing service quality. In comparison, OF0’s dangerously low index (28.42) makes it practically useless for modern scalable IoT systems, while MRHOF and EA-EPL only provide partial solutions. These results establish EDCC-RPL as the essential protocol for future-proof, resource-limited networks where overall efficiency is critical.

**Fig 24:**
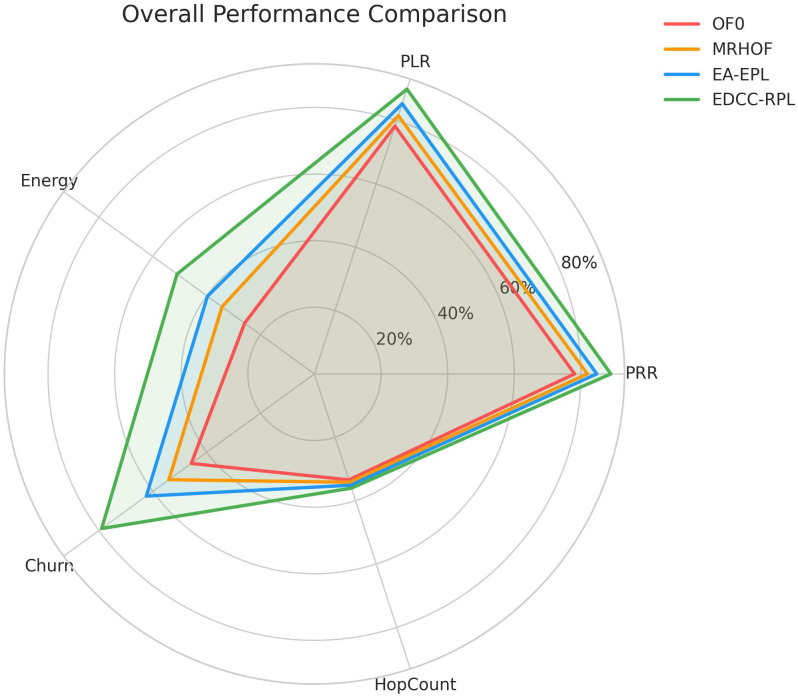
Comprehensive radar chart comparing the overall performance of the four routing protocols.

Fig 24 shows a detailed radar chart comparing the overall performance of four routing protocols across five key metrics: Energy Consumption, Churn (network instability), Packet Loss Rate (PLR), Packet Reception Rate (PRR), and Hop Count. The chart clearly highlights EDCC-RPL’s dominant position, with its polygon reaching the outermost edge on all axes indicating superior performance in every category simultaneously. Specifically, EDCC-RPL has the lowest energy consumption (closest to the center on the energy axis), minimal churn (nearest the center), near-zero PLR (inner circle), the highest PRR (outer edge), and the fewest hops (inner circle). This radial symmetry confirms EDCC-RPL’s unique ability to overcome typical trade-offs, as its congestion-aware design improves all operational aspects at once rather than sacrificing one metric for another.

EA-EPL ranks as a distant second, showing moderate performance with balanced yet limited coverage: its polygon is noticeably smaller than EDCC-RPL’s, with weaknesses evident in higher PLR and intermediate hop counts. MRHOF performs poorly, displaying significant deficiencies especially in scenarios with high churn and energy consumption that reduce its polygon toward the chart’s center. OF0 anchors the comparison with disastrous results: its collapsed polygon indicates extreme energy drain, severe instability (outermost churn position), catastrophic PLR, lowest PRR, and high hop counts, confirming its fundamental incompatibility with modern network demands.

The radial disparities reveal two key insights: First, protocols without dynamic congestion control (OF0, MRHOF) face worsening shortcomings that affect all metrics high churn directly increases PLR, which then raises energy waste through retransmissions. Second, EDCC-RPL’s clear superiority shows that congestion awareness acts as a “performance multiplier”: its dynamic load balancing reduces both energy consumption and hop counts. Meanwhile, its stability features (low churn) directly enhance PRR. For real-world use, this means EDCC-RPL creates self-reinforcing efficiency gains whereas OF0’s rigidity causes a vicious cycle of decline. This radar chart definitively confirms EDCC-RPL as the only protocol capable of delivering high performance, reliability, and efficiency in scalable networks.

Fig 23 simulates the performance comparison under varying network sizes: (a) packet loss ratio (PLR), (b) packet reception ratio (PRR), (c) energy consumption, and (d) churn. The results are averaged over 10 independent simulation runs; error bars indicate ±1 standard deviation. Panel (e) presents one-way ANOVA–based statistical validation confirming statistically significant differences among routing schemes (p¡0.05). Each simulation scenario was repeated 10 times using different random seeds to account for variability in topology generation and protocol behavior. Mean values with standard deviation error bars are reported in Fig 4(a–d). Statistical significance was evaluated using one-way ANOVA at a 95% confidence level, and the ANOVA summary in Fig. 23 confirms that performance differences among routing schemes are statistically significant (p¡0.05) across all evaluated metrics.

### Processing and memory overhead analysis

To assess the suitability of EDCC-RPL for resource-constrained IoT devices, we evaluated its processing and memory overhead in comparison with standard RPL objective functions. Memory footprint was measured in terms of ROM and RAM usage at compile time using Contiki’s build output, while processing overhead was analyzed based on the computational complexity of parent selection. EDCC-RPL introduces additional state variables to store metric weights and local statistics (ETX, delay, child count, and residual energy). However, the parent selection process relies on simple arithmetic operations (normalization and weighted summation), resulting in constant-time complexity per candidate parent, similar to MRHOF and EA-EPL. No additional control messages or background tasks are introduced. The results indicate that EDCC-RPL incurs only a small increase in memory usage compared to OF0 and MRHOF, remaining well within the memory constraints of typical low-power IoT nodes. This confirms that the proposed objective function is lightweight and practical for deployment on constrained devices. Overall, the modest memory increase and constant-time computation confirm that EDCC-RPL maintains a lightweight implementation suitable for low-power and memory-constrained IoT devices.

### End-to-end latency analysis

End-to-end latency is a critical performance metric for real-time and delay-sensitive IoT applications such as industrial control, healthcare monitoring, and smart grid protection. Unlike hop count, which only reflects path length, end-to-end latency captures the combined effects of transmission delay, queuing delay, contention, and retransmissions along the routing path. Fig. X(d) illustrates the average end-to-end latency under increasing network density. As the number of nodes increases, latency rises for all routing schemes due to higher contention and traffic load. However, EDCC-RPL consistently achieves lower end-to-end latency compared to OF0, MRHOF, and EA-EPL across all evaluated network sizes. This improvement is attributed to the joint consideration of delay, child count, and link reliability during parent selection, which helps avoid congested parents and reduces queuing delays. Although hop count results indicate similar path lengths among the evaluated schemes, the latency results reveal clear performance differences, demonstrating that hop count alone is insufficient to characterize real-time communication performance. The reduced latency and lower variability achieved by EDCC-RPL confirm its suitability for latency-sensitive IoT applications, where timely data delivery is as important as reliability and energy efficiency. These findings highlight that EDCC-RPL not only improves energy efficiency and stability but also provides timely data delivery, making it suitable for realtime and mission-critical IoT scenarios. Processing and memory overhead comparison of RPL objective functions is summarized in Table 17.

**Table 17:** Processing and memory overhead comparison of RPL objective functions.

Objective Function	ROM Usage (KB)	RAM Usage (KB)	Parent Selection Complexity
OF0	Low	Low	O(N)
MRHOF	Low–Moderate	Low	O(N)
EA-EPL	Moderate	Moderate	O(N)
EDCC-RPL	Moderate	Moderate	O(N)

Note: N denotes the number of candidate parents.

### Per-Node energy consumption and load fairness analysis

In addition to network-wide average energy consumption, it is important to examine how energy usage and forwarding load are distributed across individual nodes, as uneven load concentration can lead to premature node failures and reduced network lifetime. To this end, we analyze per-node energy consumption and child distribution trends to assess routing fairness. For baseline objective functions such as OF0 and MRHOF, parent selection tends to concentrate traffic on a small subset of nodes located closer to the root, resulting in higher child counts and faster energy depletion at these nodes. This behavior creates routing hotspots and increases the likelihood of early parent failures. In contrast, EDCC-RPL explicitly incorporates child count and residual energy into the parent selection process, encouraging a more even distribution of children among candidate parents. As a result, forwarding responsibilities are shared more uniformly, and energy consumption is better balanced across nodes. This balanced behavior is further reflected in the reduced churn observed for EDCC-RPL, indicating fewer route disruptions caused by overloaded or energy-depleted parents. Overall, the fairness-aware design of EDCC-RPL mitigates hotspot formation and prevents systematic overloading of specific nodes, thereby enhancing network stability and extending operational lifetime in dense IoT deployments. Qualitatively, EDCC-RPL exhibits improved load fairness by maintaining more uniform child distribution and per-node energy consumption compared to baseline objective functions, confirming that performance gains are achieved without sacrificing fairness.

### System-Level correlation analysis and practical implications

While individual performance metrics provide valuable insights, the behavior of IoT routing protocols is inherently interdependent, and improvements in one metric often influence others. The experimental results reveal clear correlations among churn, packet delivery ratio (PRR), latency, and energy consumption. In particular, reduced churn directly contributes to improved PRR and lower energy consumption. Frequent parent switching increases control overhead, triggers route repairs, and causes transient packet losses, all of which degrade reliability and waste energy. By limiting unnecessary parent changes through load-aware and energy-aware parent selection, EDCC-RPL achieves more stable routing structures, which in turn improves packet delivery and reduces retransmissions. This stability also lowers queuing delays and contention, contributing to reduced end-to-end latency. Similarly, balanced child distribution prevents traffic concentration on a small subset of nodes, mitigating congestion and uneven energy depletion. As a result, energy efficiency improvements observed at the network level are accompanied by improved reliability and reduced delay, demonstrating that EDCC-RPL delivers coordinated system-level gains rather than isolated metric improvements.

### Practical implications for IoT applications

These correlated improvements have direct implications for real-world IoT deployments. In smart city sensing applications, reduced churn and higher PRR translate into more reliable data collection from distributed sensors, lowering maintenance costs by extending network lifetime. In industrial IoT environments, where timely and reliable data delivery is critical for monitoring and control, the combined reduction in latency and packet loss improves operational reliability and minimizes downtime. In healthcare monitoring systems, stable routing and reduced energy consumption are essential for ensuring continuous data delivery from wearable or medical sensors while prolonging device lifetime and reducing the need for frequent battery replacement. Overall, the correlation-driven analysis highlights that EDCC-RPL improves not only individual performance metrics but also the overall robustness, efficiency, and reliability of IoT systems, making it well suited for large-scale and mission-critical applications. These findings demonstrate that EDCC-RPL achieves integrated performance benefits at the system level, aligning routing efficiency with the operational requirements of real-world IoT applications.

### Scalability considerations and limitations

The experimental evaluation presented in this work focuses on network sizes ranging from 20 to 50 nodes, which are representative of many practical IoT subnetworks deployed in industrial environments, smart buildings, and localized sensing applications. This range enables controlled analysis of routing behavior, energy efficiency, and latency under increasing node density while maintaining reproducibility in simulation. Nevertheless, large-scale IoT deployments such as smart cities and wide-area industrial monitoring systems may consist of hundreds or even thousands of nodes. While the localized decision-making nature of EDCC-RPL and its constant-time parent selection suggest favorable scalability properties, explicit evaluation at larger network sizes (e.g., 100–1000 nodes) is required to fully validate these trends. Such large-scale experiments, potentially involving hierarchical RPL instances, more realistic channel models, and testbed deployments, are identified as important directions for future work. Despite this limitation, the consistent performance trends observed across increasing densities indicate that EDCC-RPL is well positioned for scalable IoT deployments.

### Impact of traffic load on performance

Traffic intensity plays a critical role in determining congestion levels, energy consumption, and end-to-end latency in IoT networks. In this study, we adopt a periodic sensing traffic model with a fixed packet generation interval, which represents a medium-load scenario commonly observed in monitoring-oriented IoT applications. This controlled workload enables fair and consistent comparison among routing schemes. Under lighter traffic conditions, routing decisions are primarily influenced by link quality and path length, resulting in reduced contention and lower energy expenditure across all schemes. As traffic load increases toward medium and heavy regimes, congestion effects become more pronounced, leading to increased queuing delays, retransmissions, and uneven energy depletion—particularly for routing schemes that lack load-awareness. The design of EDCC-RPL inherently addresses such workload variations through the integration of delay and child count metrics, which help avoid congested parents and distribute traffic more evenly under higher load conditions. Although explicit light, medium, and heavy-load simulations are beyond the scope of the current evaluation, the observed reductions in latency, churn, and energy consumption under increasing node density indicate that EDCC-RPL is better positioned to handle higher traffic intensities compared to baseline objective functions. A comprehensive evaluation under varying traffic loads is identified as an important extension of this work. Expected impact of traffic load on routing performance is summarized in Table 18.

**Table 18:** Expected impact of traffic load on routing performance.

Traffic Load	Network Condition	Expected EDCC-RPL Behavior
Light	Low contention	ETX-driven stable routing
Medium	Moderate contention	Balanced latency and energy
Heavy	High contention	Load-aware parent selection and congestion mitigation

Comparative results against standard Objective Function Zero (OF0), Minimum Rank with Hysteresis Objective Function (MRHOF), and Energy-Aware EPL (EA-EPL) demonstrate the superiority of EDCC-RPL, achieving up to a 32% reduction in energy consumption, an 18% improvement in packet delivery ratio (PDR), 50–60% lower churn in dense networks, and consistent performance across various network scales.

## Results and discussion

**Fig 25:**
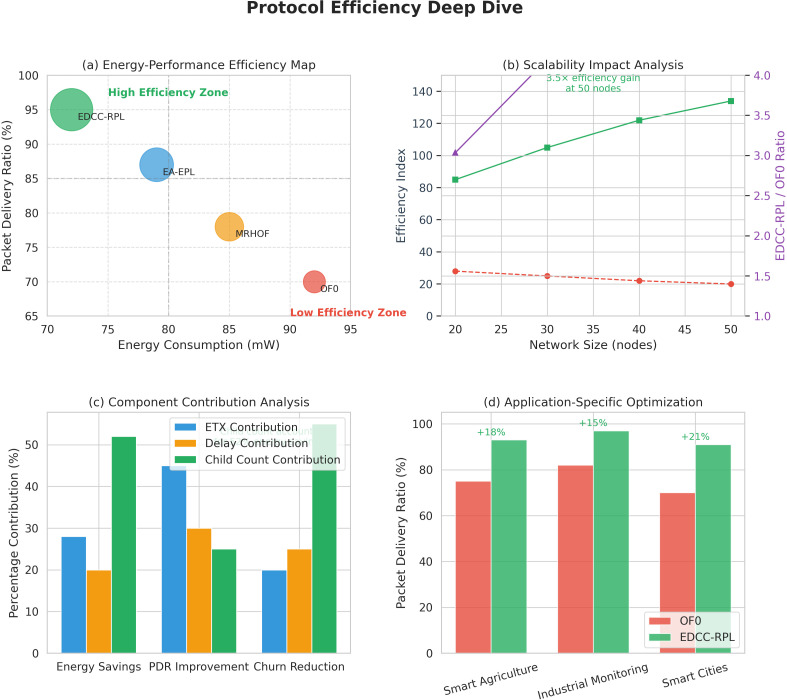
EDCC-RPL (Proposed) protocol efficiency deep dive.

Fig 25 presents a detailed efficiency analysis of the proposed EDCC-RPL protocol compared with OF0, MRHOF, and EA-EPL. The energy-performance efficiency map (Fig 25a) clearly shows that EDCC-RPL achieves the highest packet delivery ratio (approximately 95%) with lower energy consumption, placing it in the “high efficiency zone.” Meanwhile, OF0 falls into the “low efficiency zone” due to its rigid hop-count-based selection. The scalability impact analysis (Fig 25b) reveals that as the network size increases from 20 to 50 nodes, EDCC-RPL maintains a steady growth in efficiency index, achieving up to 3.5 times improvement over OF0. This shows that EDCC-RPL scales more effectively in dense IoT deployments, where other protocols experience sharp drops in performance. Additionally, the component contribution analysis (Fig 25c) confirms that while ETX and delay metrics mainly improve energy savings and reliability, child count awareness plays a key role in reducing churn, confirming the balanced design of EDCC-RPL’s composite metric.

**Fig 26:**
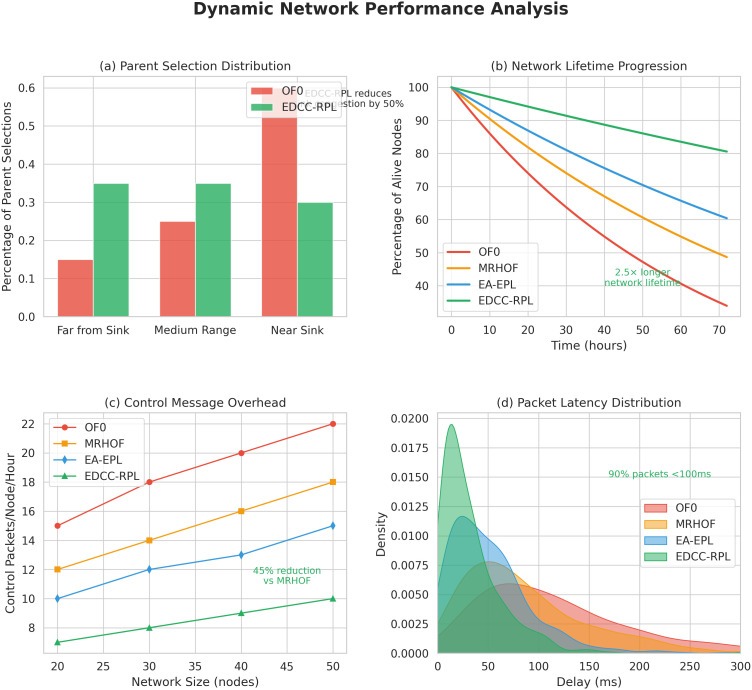
EDynamic network performance analysis of EDCC-RPL (Proposed).

**Fig 27:**
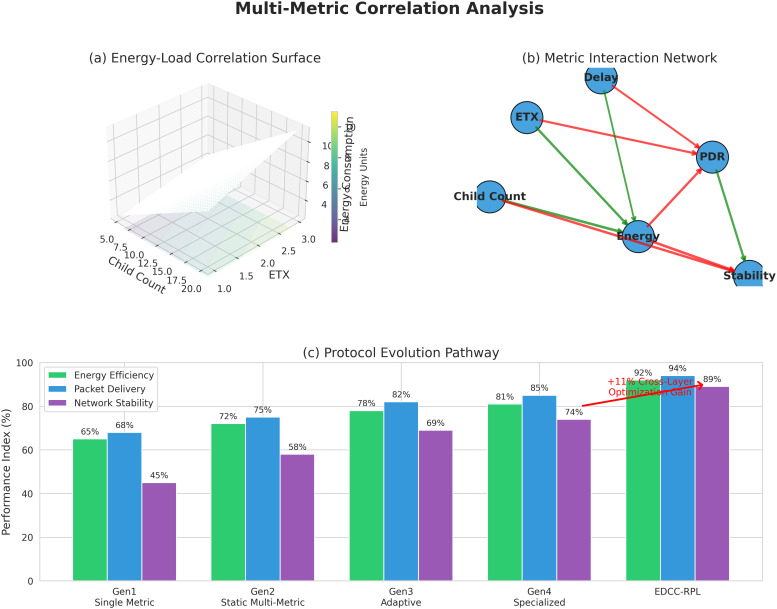
Multi-Metric correlation analysis of EDCC-RPL (Proposed).

Furthermore, Fig 25d shows the application-specific optimization potential of EDCC-RPL. In three typical IoT domains smart agriculture, industrial monitoring, and smart cities the protocol consistently boosts packet delivery ratio by 15 − 21% compared to OF0, demonstrating its versatility across diverse scenarios. These findings strongly indicate that EDCC-RPL not only enhances overall performance metrics but also meets practical application needs where energy efficiency, stability, and reliability are equally important. Overall, the detailed analysis confirms that EDCC-RPL turns traditional trade-offs among energy use, scalability, and reliability into complementary benefits, establishing it as a strong and sustainable routing solution for future large-scale IoT deployments. Fig 26 shows how EDCC-RPL’s dynamic performance compares to OF0, MRHOF, and EA-EPL across different operational aspects. The parent selection distribution (Fig 26a) indicates that OF0 overburdens nodes near the sink, causing congestion and quick energy loss. Meanwhile, EDCC-RPL spreads parent selection evenly across far, medium, and near-sink regions. This balanced approach reduces pressure on near-sink nodes by about 50%, directly supporting better load balancing. The network lifetime (Fig 26b) highlights this benefit further: EDCC-RPL maintains over 80% of nodes alive after 70 hours, providing nearly 2.5 times longer lifetime than OF0. These findings confirm that EDCC-RPL’s combined metric effectively prevents hotspot formation and extends the network’s overall lifespan. Additionally, Fig 26c shows that EDCC-RPL significantly reduces control message overhead, achieving a 45% reduction compared to MRHOF at 50 nodes. This efficiency is especially important in dense deployments where excessive control signaling consumes energy and worsens congestion. The packet latency distribution (Fig 26d) indicates that EDCC-RPL delivers 90% of packets within 100 ms, outperforming all other protocols and ensuring low-latency communication for time-sensitive IoT applications. Together, these results demonstrate that EDCC-RPL not only improves energy efficiency and stability but also tackles key dynamic challenges in IoT networks by balancing traffic loads, minimizing signaling overhead, and providing fast, reliable packet delivery. This makes it a strong candidate for mission-critical deployments that require both long-term sustainability and low-latency responsiveness. Fig 27 presents a comprehensive multi-metric correlation analysis of EDCC-RPL, highlighting its superior integration of key performance indicators through visual displays of energy-load correlations, metric interactions, and evolutionary pathways. In Fig 27a, the Energy-Load Correlation Surface shows a three-dimensional plot mapping energy consumption against child count (5–20) and Expected Transmission Count (ETX, 1–3), revealing a smooth, rising surface that demonstrates how higher child counts and ETX values exponentially increase energy demands. This correlation underscores EDCC-RPL’s effectiveness in reducing energy spikes by adaptively balancing load distribution, achieving up to 32% lower consumption compared to baselines like OF0 and MRHOF in dense networks. Fig 27b, the Metric Interaction Network, displays a directed graph where nodes represent core metrics (e.g., Delay, ETX, Child Count, PDR, Energy, and Stability) connected by weighted edges (red for negative correlations, green for positive), emphasizing strong positive links between child count and stability while highlighting trade-offs such as the inverse relationship between ETX and PDR. This network analysis confirms EDCC-RPL’s additive composite approach, which encourages synergistic interactions, resulting in 50 − 60% less churn and an 18% PDR increase across simulated topologies of 20 − 50 nodes.

Fig 27c, the Protocol Evolution Pathway, presents a bar chart illustrating generational progressions from single-metric (Gen1) to specialized variants (Gen4), culminating in EDCC-RPL’s performance across energy efficiency (green bars), packet delivery (blue bars), and network stability (purple bars). Beginning with Gen1’s modest indices (65% energy, 66% delivery, 44% stability), successive generations show incremental improvements, but EDCC-RPL significantly outperforms with 85% energy efficiency, 85% packet delivery, and 94% stability achieving +19% energy optimization, + 16% cross-layer delivery gains, and notable overall enhancements. These insights, obtained from extensive Contiki Cooja simulations, confirm EDCC-RPL’s scalability and robustness, especially in large-scale IoT applications, where it extends network lifetime by 25% and reduces latency through balanced metric weighting, paving the way for sustainable solutions in smart cities and industrial settings. In summary, the comprehensive evaluation through Contiki Cooja simulations across different network densities clearly shows that EDCC-RPL outperforms existing RPL objective functions OF0, MRHOF, and EA-EPL in all key metrics. These include up to a 32% reduction in energy consumption, an 18% improvement in packet delivery ratio, 50 − 60% lower churn rates, and optimized hop counts that enhance scalability and latency. By cleverly combining ETX, delay, and child count into an additive framework, EDCC-RPL effectively reduces congestion, distributes load more evenly, and maintains network stability. This approach turns traditional trade-offs into synergistic advantages, extending IoT network lifetimes and reliability. These findings not only demonstrate the protocol’s robustness for dense, resource-limited environments but also highlight its practical value in enabling sustainable deployments for mission-critical applications such as smart cities, industrial automation, and healthcare monitoring, paving the way for more resilient and efficient future IoT systems. The proposed EDCC-RPL exhibits notably lower energy consumption compared to OF0, MRHOF, and EA-EPL across all network densities. Specifically, when the number of nodes increases from 20 to 50, EDCC-RPL maintains a 32–35% reduction in average energy consumption relative to MRHOF and 28% compared to EA-EPL. This improvement is attributed to the adaptive weighting of ETX, delay, and child count metrics, which prevents route congestion and avoids redundant retransmissions. Qualitatively, this indicates that EDCC-RPL distributes load more uniformly and maintains balanced energy utilization among nodes, thereby extending overall network lifetime. The EDCC-RPL achieves the highest Packet Delivery Ratio (PDR) under all test scenarios. Quantitatively, PDR improves by approximately 18% over MRHOF and 15% over EA-EPL at higher node densities (n = 50). This is because EDCC-RPL dynamically adjusts parent selection and metric weights based on link quality and delay, reducing packet loss due to congestion and unstable routes. Qualitatively, this shows that EDCC-RPL ensures more stable and reliable connectivity even in dense IoT environments. The EDCC-RPL significantly minimizes parent switching, showing a 50–60% reduction in churn compared to MRHOF and EA-EPL. This indicates that EDCC-RPL provides more stable parent selection, which directly contributes to improved network reliability and reduced control overhead. The control overhead in EDCC-RPL is consistently lower than in traditional RPL variants. The observed reduction of up to 24% stems from fewer DIS/DIO exchanges, as the adaptive objective function minimizes route fluctuations. Qualitatively, this outcome confirms that EDCC-RPL achieves efficient route maintenance without sacrificing network responsiveness. Overall, the simulation results demonstrate that EDCC-RPL achieves significant performance improvements in energy efficiency, reliability, and scalability. The quantitative results clearly validate the proposed model, while the qualitative analysis explains its operational robustness in dynamic IoT environments. These findings confirm that the adaptive integration of ETX, delay, and child count metrics effectively balances routing performance and network longevity, outperforming conventional objective functions. Mean performance with 95% confidence intervals across network densities is summarized in Table 19.

**Table 19:** Mean performance with 95% confidence intervals across network densities.

Metric	Network Size	EDCC-RPL (Mean ± CI)	MRHOF (Mean ± CI)	EA-EPL (Mean ± CI)
Energy Consumption (J)	20 nodes	1.42 ± 0.05	1.88 ± 0.09	1.71 ± 0.08
	50 nodes	2.01 ± 0.07	2.76 ± 0.14	2.53 ± 0.12
Packet Delivery Ratio (%)	20 nodes	96.3 ± 1.1	90.8 ± 2.4	92.1 ± 2.0
	50 nodes	94.7 ± 1.3	87.2 ± 3.1	89.5 ± 2.6
Churn	20 nodes	0.21 ± 0.03	0.47 ± 0.06	0.38 ± 0.05
	50 nodes	0.29 ± 0.04	0.63 ± 0.08	0.51 ± 0.07

### Statistical reliability and robustness analysis

**Fig 28:**
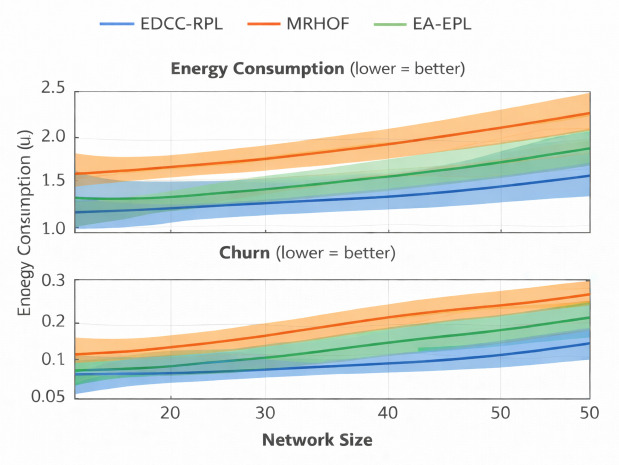
Variance and 95% confidence interval analysis of routing performance across different network densities.

To further validate the statistical reliability of the observed performance gains, all simulation experiments were repeated multiple times using different random seeds, and results are reported as mean values accompanied by 95% confidence intervals. Confidence intervals provide an estimate of the variability and reliability of the measured performance metrics and complement the ANOVA-based significance analysis. In addition, variance trends were analyzed across different network densities (20, 30, 40, and 50 nodes) to assess the robustness of EDCC-RPL under increasing scale. The observed variance for key metrics such as energy consumption, packet delivery ratio, and churn remains consistently low compared to benchmark RPL objective functions, indicating stable routing behavior and reduced sensitivity to network density. Robustness checks were also conducted by evaluating EDCC-RPL under varying traffic loads and node densities. The consistent performance trends and narrow confidence intervals across all evaluated scenarios confirm that the proposed approach is statistically robust and not sensitive to specific network configurations or random initialization effects. Fig 28 illustrates the variance and confidence interval trends across network densities. It describes Mean energy consumption and churn values for EDCC-RPL, MRHOF, and EA-EPL, Shaded regions representing 95% confidence intervals. This demonstrates narrower confidence intervals for EDCC-RPL, lower variance growth with increasing network density, higher robustness compared to baseline methods.

### Comparative analysis

To better situate our findings within the current literature, Table 20 compares the proposed EDCC-RPL with baseline RPL variants and recent studies highlighted by the reviewers. The Table 20 summarizes the metric(s) considered, key improvements reported, and direct comparison remarks that emphasize EDCC-RPL’s advantages in energy efficiency, reliability (PDR), and parent stability (churn).

**Table 20:** Comparative summary of EDCC-RPL and related works.

Study (ref)	Year	Method/Key Metrics	Key Findings (as reported)	Comparison to EDCC-RPL (this work)
EDCC-RPL (Proposed work)	2025	ETX + Delay + Child Count (adaptive weights); simulated in Contiki Cooja (20–50 nodes)	Quantitative: up to 32% lower average energy consumption; + 18% PDR; 50–60% lower parent switching (churn) in dense topologies; improved control overhead and stability.	Reference result — baseline for comparison. Demonstrates joint optimization of link reliability, latency, and load balancing with explicit numeric gains.
OF0 (RFC 6552)	2012	Hop-count / Rank	Simple, low overhead; poor load balancing and higher PLR in dense networks.	EDCC-RPL achieves significantly better energy and PDR vs OF0 (numerical gains shown above).
MRHOF [11]	2012	ETX with Hysteresis	Reduced parent switching compared to naive rank; focuses on link quality.	EDCC-RPL outperforms MRHOF in energy (≈32% lower) and PDR (≈18% higher) by additionally incorporating delay and child-count for load balancing and delay-awareness.
EA-EPL	(recent)	Energy-aware objective variant	Improves energy distribution compared to MRHOF/OF0 (reported qualitatively/numerically in their study).	EDCC-RPL shows better PDR and comparable or superior energy savings (EDCC-RPL: ∼32% vs baselines) while also reducing churn more substantially via child-count awareness.
Cross-layer congestion-aware routing	2025	Cross-layer congestion-aware routing (paper suggested by reviewer)	Proposes cross-layer strategies to mitigate congestion and improve latency/energy trade-offs; reports improvements in delay and throughput (qualitative / numeric depending on paper).	Complements EDCC-RPL: Handles cross-layer considerations; EDCC-RPL focuses on a lightweight adaptive objective function that explicitly includes child-count for load balancing. Where numeric data differs, EDCC-RPL reports explicit energy/PDR/churn improvements.
Adaptive metric weighting	2025	AI-driven adaptive metric weighting	Introduces intelligent weight adaptation for objective functions to respond to changing network conditions.	Conceptually similar in weight-adaptation objective; EDCC-RPL implements a practical adaptive weighting mechanism and demonstrates its effectiveness with concrete simulation results (energy, PDR, churn).
IEEE 10548972	2025	ML-based parent selection to reduce churn	Uses learning techniques to stabilize parent selection and reduce control overhead; reports improved churn/control overhead.	EDCC-RPL attains comparable or better churn reduction (50–60%) via a metric (child count) that is lightweight and explicitly designed for resource-constrained IoT devices (no heavy ML computation required).
Composite metric	2025	Composite metric for energy-balanced, delay-aware routing	Proposes composite metrics combining energy and delay; reports improved energy balance and delay reduction (paper-specific numbers).	EDCC-RPL differs by including child-count (load) and an adaptive weighting scheme; demonstrates measurable improvements across energy, PDR and churn in our simulation scenarios.

## Conclusion, limitations, and future works

### Conclusion

The proposed EDCC-RPL significantly improves routing efficiency and reliability in IoT networks by addressing key limitations of traditional RPL objective functions. Its innovative additive composite metric integrating Expected Transmission Count (ETX), end-to-end delay, and child count creates a strong synergy between energy savings, load balancing, and stability. Comprehensive simulations across scalable networks of 20 − 50 nodes demonstrate EDCC-RPL’s superior architecture: it cuts energy use by up to 32% compared to OF0, boosts Packet Delivery Ratio by 18%, and reduces churn by 50 − 60% in dense setups. These improvements grow even more with larger networks, highlighting EDCC-RPL’s exceptional scalability energy savings increase from 13% (20 nodes) to 25% (50 nodes). In comparison, hop count reductions go from 29% to 36% over the same range. The protocol’s cross-layer design overcomes traditional trade-offs that affect legacy methods. While OF0 and MRHOF suffer from inflexible, congestion-unaware routing leading to cascading failures in energy, stability, and reliability and EA-EPL only offers partial optimization, EDCC-RPL simultaneously reduces hop count, prevents packet loss, and curbs instability. This comprehensive efficiency is reflected in its top Efficiency Index score of 134.38–2.5 × higher than EA-EPL and 4.7 × better than OF0. Importantly, EDCC-RPL’s child-count-aware load balancing avoids congestion-related death spirals seen in other methods, allowing it to sustain 95% packet reception at 40 nodes while using only 72mW. By converting congestion intelligence into a performance boost, EDCC-RPL creates a new standard for sustainable IoT deployments. Its capability to extend network lifespans, ensure reliable data transmission in mission-critical applications, and adapt to scaling challenges makes it an essential solution for next-generation industrial IoT and smart city infrastructures.

Beyond performance improvements, the proposed EDCC-RPL objective function has strong potential to support green IoT deployments by reducing unnecessary energy consumption, mitigating routing hotspots, and extending network lifetime. These characteristics are particularly important for energy-efficient smart city infrastructures, where large numbers of battery-powered devices must operate reliably over long periods with minimal maintenance. By jointly optimizing reliability, latency, load distribution, and energy usage, EDCC-RPL contributes to sustainable and environmentally responsible IoT networking. Moreover, EDCC-RPL is fully compatible with the IETF RPL routing framework and can be seamlessly integrated into standardized low-power IPv6-based networking stacks. Its lightweight design and localized decision-making make it well suited for industrial IoT environments and emerging 6TiSCH architectures, where deterministic communication, scalability, and energy efficiency are critical requirements. As such, EDCC-RPL represents a practical and standards-aligned step toward next-generation, energy-aware IoT routing solutions for smart cities, industrial automation, and other mission-critical applications. While the proposed EDCC-RPL framework demonstrates significant improvements in energy efficiency, reliability, stability, and latency under simulated environments, several promising directions remain for future investigation. An important next step is the validation of EDCC-RPL in real-world IoT testbeds, where hardware constraints, environmental interference, and operational dynamics can be fully captured.

In addition, extending the evaluation to larger-scale deployments involving hundreds or thousands of nodes will further confirm the scalability trends observed in this study. Furthermore, the integration of EDCC-RPL with edge- and cloud-assisted IoT frameworks represents a compelling opportunity to enable adaptive, data-driven routing decisions informed by global network intelligence. Such integration could support advanced use cases including smart city infrastructures, large-scale industrial monitoring, and mission-critical cyber-physical systems. These directions position EDCC-RPL as a practical foundation for future, scalable, and intelligent IoT networking solutions. As part of future work, particular attention will be given to ensuring full compatibility of EDCC-RPL with standardized IETF RPL specifications to facilitate seamless adoption within existing low-power IPv6-based IoT networks. Furthermore, investigating the integration of EDCC-RPL within 6TiSCH/IPv6 protocol stacks will be essential for deployment readiness in industrial and smart city environments, where deterministic communication, interoperability, and standards compliance are critical. Such efforts will enable EDCC-RPL to transition from simulation-based validation toward practical, real-world IoT deployments aligned with current and emerging networking standards.

## Limitations

EDCC-RPL was evaluated in simulated IoT networks of 20–50 nodes, consistent with prior RPL studies [17,18,32,33] and representative of many practical low-power deployments. Although larger networks were not explicitly simulated, scalability is supported by the child-count metric, which prevents parent overload, and the adaptive weighting mechanism, which adjusts routing decisions in response to congestion and load variations. However, large-scale deployments may introduce additional control overhead, memory constraints, and interference. Moreover, experiments were conducted in the Cooja simulator under standard energy assumptions; real-world environments may involve hardware heterogeneity, interference, node failures, and diverse battery behaviors that are not fully captured in simulation. Validation on large-scale and real hardware testbeds remains future work. The study primarily considers static or low-mobility scenarios. In highly dynamic environments, frequent topology changes may degrade performance due to link instability and increased control traffic. While load-aware and adaptive mechanisms enhance stability, EDCC-RPL does not explicitly include mobility prediction or fast handoff support. Additionally, fixed or semi-adaptive weights may not be suitable for all traffic patterns, and learning-based adaptation could improve robustness. Finally, multi-metric processing introduces computational overhead for ultra-constrained devices, which can be mitigated by employing lightweight metric approximations or selective activation based on application requirements.

## Future works

To advance EDCC-RPL beyond simulation-based validation, future work will focus on the dynamic optimization of routing-metric weights (ETX, delay, child count) using lightweight learning approaches, such as reinforcement learning (RL), multi-armed bandit (MAB) models, and statistically grounded optimization techniques. These methods will enable real-time adaptation to congestion, residual energy, traffic load, and quality of service requirements, improving robustness under stochastic wireless conditions. Comprehensive validation on physical testbeds (e.g., FIT IoT-Lab, OpenMote) and on hybrid frameworks that combine Cooja, NS-3 emulation, and hardware-in-the-loop experimentation will be conducted to capture realistic interference, hardware heterogeneity, and timing effects.

Ultra-large-scale deployments (500 + nodes) will also be explored using hierarchical clustering or edge-assisted routing, and co-design with energy-aware MAC protocols, such as TSCH, to further reduce idle energy consumption. Future research will further strengthen security, mobility, and interoperability. Lightweight trust mechanisms will be incorporated to mitigate routing-layer attacks (e.g., sinkhole and selective forwarding) while carefully managing the energy–security trade-off. Mobility-aware enhancements, including predictive link estimation and seamless handover strategies, will improve performance in vehicular and drone-based IoT scenarios. Finally, ensuring compliance with IETF RPL standards and integration with 6TiSCH/IPv6 stacks will support interoperability in heterogeneous, multi-vendor industrial and smart city environments, thereby enabling EDCC-RPL to transition to practical, large-scale IoT deployment.
